# Accessory respiratory muscles performance among people with spinal cord injury while singing songs with different musical parameters

**DOI:** 10.1371/journal.pone.0305940

**Published:** 2024-07-05

**Authors:** Muhammad Imran Ramli, Nur Azah Hamzaid, Julia Patrick Engkasan, Juliana Usman, Marzelan Salleh, Wee Duen Hueh

**Affiliations:** 1 Department of Biomedical Engineering, Faculty of Engineering, Universiti Malaya, Kuala Lumpur, Malaysia; 2 Department of Rehabilitation Medicine, Faculty of Medicine, Universiti Malaya, Kuala Lumpur, Malaysia; 3 Department of Music, Faculty of Creative Arts, Universiti Malaya, Kuala Lumpur, Malaysia; Lahore University of Biological and Applied Sciences, PAKISTAN

## Abstract

People with spinal cord injury (SCI) experience respiratory dysfunctions which include hypersecretions, bronchospasm, and respiratory muscles weakness. Singing therapy has been implemented as part of respiratory muscle training (RMT) to improve their muscle strength. Singing different types and genres of songs may elicit specific recruitment of respiratory muscles, attributed to the variation of the songs’ characteristics including tempo, pitch, and rhythmic complexity. This study aims to determine the effect of singing songs with different characteristics on the accessory respiratory muscle performance among people with SCI. Thirteen male SCI participants of ASIA A and B (C4 –T11) were recruited. Respiratory muscle signals were retrieved by placing two mechanomyography (MMG) sensors on the sternocleidomastoid (SCM) and rectus abdominis (RA) muscles. Eight music experts categorized several songs into four categories based on their pitch, tempo, and rhythmic complexity. Each participant sang one song from each category. Findings showed statistically significant difference in RA and SCM responses among all categories (P < 0.01). The SCM muscle is most active while singing high pitch songs. While the RA is most active during slow tempo and easy rhythmic complexity. This shows that different accessory respiratory muscle is activated by people with SCI while singing songs with different characteristics. Clinicians could benefit from this knowledge while prescribing singing therapy or exercise among people with SCI in the future.

## Introduction

During quiet breathing, the inspiratory muscles (diaphragm and external intercostals) expand the thoracic cavity causing air to fill the lungs. This is followed by passive recoil of the chest wall during expiration. However, during heightened physical activities, accessory respiratory muscles are activated to keep up with the higher oxygen demand. These muscles include sternocleidomastoid, the scalenus, latissimus dorsi, and the abdominal muscles.

People with spinal cord injury (SCI) often have symptoms of respiratory insufficiency, particularly those with injury involving the cervical and thoracic region [1, 2]. The higher the level of lesion, the more respiratory muscles are impaired causing significant respiratory impairments. After SCI, the motor pathways that innervate the muscles of respiratory are disrupted, leading to muscle weakness, spasticity, and paralysis [3]. People with SCI also suffer from impairment of respiratory motor control [4], limiting the recovery [5, 6] and quality of life [7].

Currently, clinicians and researchers have adopted several exercises to improve the respiratory muscles strength among people with SCI. This exercise is called respiratory muscle training (RMT) and includes exercises for inspiration, expiration or both of a certain duration and intensity to improve the strength and endurance of the muscles of respiration [8]. RMT includes resistive load training [9–12], pressure threshold loading training [13–16], normocapnic hyperpnea training [17–20], and singing training [21].

Utilizing singing as a training strategy to improve muscle strength among people with SCI is relatively new among researchers and clinicians compared to other conventional exercises. Singing has been shown to improve the respiratory strength, endurance and quality of life in many populations, such as degenerative speech/voice disorder [22–25], chronic obstructive pulmonary disease (COPD) [26], and cancer patients [27, 28]. Like any other RMT, singing requires repeated and extensive inhalation and exhalation, leading to improvement in respiratory function [8].

In classical and trained singers, studies have shown that different muscles of respiration were recruited at different musical parameters [29–32]. Other than pitch, tempo and rhythmic complexity also affect the breathing mechanism while singing. Pitch refers to the control of the fundamental frequency of voice. The higher the pitch, the higher the tone of voice. Tempo refers to the speed of song in beats per minute (BPM). The faster the tempo, the faster the speed of song. Meanwhile, rhythmic complexity refers to complexity of the rhythm of sung syllables. Rapid changes in the syllables of a song and complex or syncopated rhythm make it more difficult to sing.

Currently there are limited studies implementing singing training among people with SCI. Tamplin et al. (2013) have established trends of improvements in speech intensity, respiratory function, and muscle strength after singing a familiar song [21]. However, no study has investigated the effect of singing different characteristics of song among people with SCI. This knowledge is useful for clinical application with regards to the selection of appropriate songs for RMT. Therefore, the study aims to determine the effect of singing at different tempo, pitch and rhythmic complexity on the accessory respiratory muscle activity while singing among people with SCI.

## Materials and methods

In this study, MMG (BIOPAC MP150 system with 2-channel vibromyography) data was monitored and measured from the sternocleidomastoid (SCM) and rectus abdominis (RA) muscles while singing.

### Subjects

A total of 13 individuals with SCI (13 males) were recruited in this study (Table 1). All participants were recruited between 3^rd^ June 2022 until 10^th^ August 2022. The authors had access to information that could identify individual participants during or after data collection. The authors obtained written consent from each participant before the experiment began. The inclusion criteria include SCI level between C4 and T11; American Spinal Injury Association Impairment Scale (AIS) A or B; aged 18 years and above. People with SCI above C4 level were not included to ensure all included participants’ diaphragm innervation is still intact. Meanwhile, volunteers with injury level below T11 were not eligible because there is minimal respiratory muscle impairment below this level of injury. AIS A or B indicates the completeness of neurological deficits; A indicates complete motor and sensory injury whilst B indicates incomplete sensory. The exclusion criteria include any other disorders that could affect respiratory functions such as heart failure, coronavirus disease (COVID-19), and tuberculosis. Any volunteers who were unable to sing due to vocal cord conditions or sore throat were also excluded from this study.

**Table 1:** Participant demographics.

Demographic	SCI participants (n = 13)
Age (year)	48.8 ± 8.6
Height (m)	1.6 ± 0.1
Weight (kg)	67.3 ± 11.6
BMI (kg/m^3^)	24.9 ± 4.0

Note: BMI = body mass index

### Study protocol

The data was collected with SCI participants in a lab in the Department of Biomedical Engineering, Universiti Malaya, Kuala Lumpur. In addition, some data were also monitored and recorded in a non-governmental organization (NGO) building named Beautiful Gate Foundation for the Disabled in Kepong, Kuala Lumpur. The University of Malaya Research Ethics Committee (Ref: UM.TNC2/UMREC– 638) approved the research ethics clearance before the protocol was conducted. Each participant provided informed consent prior to joining the experiment.

Researchers have collectively established MMG as a tool to demonstrate skeletal muscle performance through vibration in various clinical settings [33–35]. Studies using alternative devices in measuring the mechanical activities of the respiratory muscles such as MMG are imperative to complement electromyography (EMG) assessments [33]. According to some researchers, a signal response that is inherently mechanical would provide a more representative model of muscle contraction, as this contraction is fundamentally driven by the mechanical properties of the muscle fibre [36].

Two MMG sensors were attached to participants’ SCM and RA muscles to observe accessory respiratory muscle activity while singing. These two muscles were chosen because they are both superficial, which makes them palpable thus able to be located and captured by MMG. The MMG sensor on SCM was placed based on palpation of the muscle belly during manually resisted neck flexion contractions [37]. The sensor on RA was placed 2 cm lateral to the umbilicus, over the muscle mass [38].

### Song categorization

**Fig 1:**
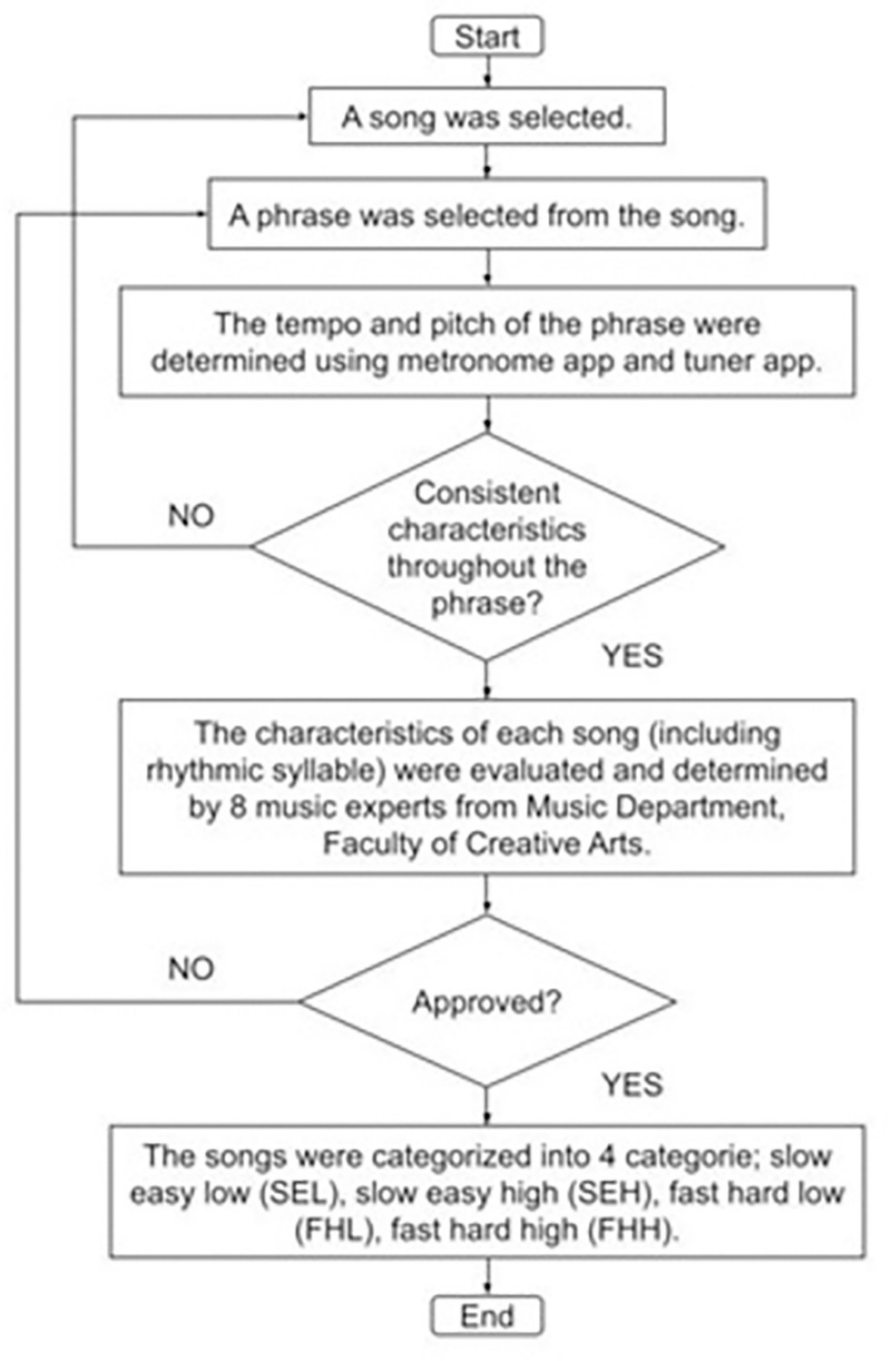
The process behind the categorization and characterization of the songs.

In this study, 4 songs were chosen by the SCI participants from 4 different categories, in terms of their tempo, pitch and rhythmic complexity (Fig 1). The participants chose one song from each category from a total of 48 songs (S4 Appendix). Eight music experts from the Department of Music, Faculty of Creative Arts, Universiti Malaya were recruited to evaluate the characteristics of the songs (S1 and S4 Appendices). A phrase with consistent tempo, pitch and rhythmic complexity for each song were identified. Before singing, MMG signal during inspiration and expiration without singing was recorded.

**Fig 2:**
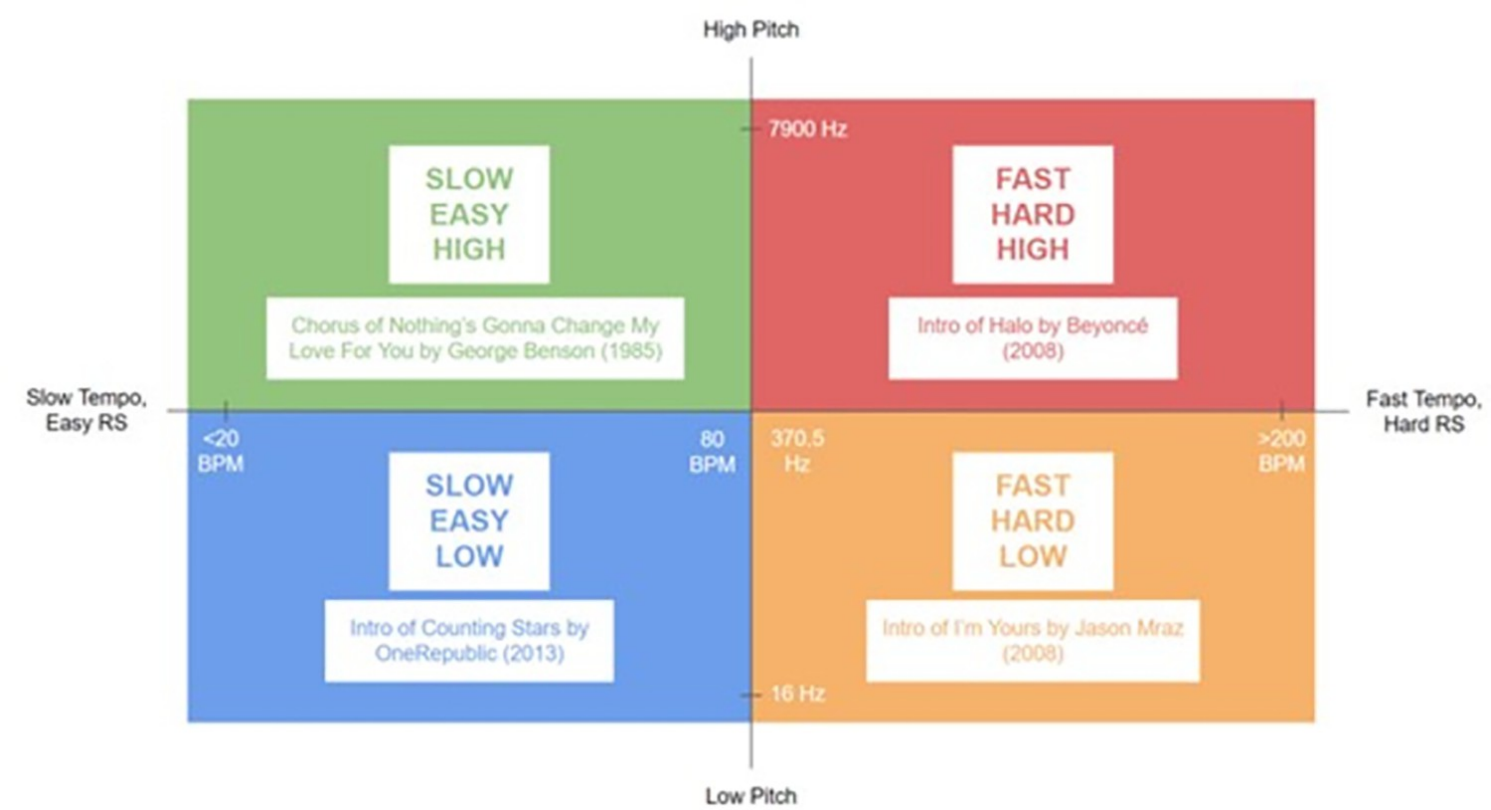
The categories of song including examples. Note: RS = rhythmic complexity; BPM = beats per minute.

All participants were required to choose one song from each category: (i) slow tempo easy low pitch (SEL), (ii) slow tempo easy high pitch (SEH), (iii) fast tempo hard low pitch (FHL) and (iv) fast tempo hard high pitch (FHH) (Fig 2). ‘Slow’ and ‘fast’ refer to the tempo of the song (S3 Appendix). ‘Easy’ and ‘hard’ refer to the rhythmic complexity. Meanwhile, ‘low’ and ‘high’ refer to the pitch of the song (S2 Appendix).

There were no specific instructions on how they were supposed to sing the song, except to follow the musical cues from the pre-recorded track. Every participant used headphones while listening to and singing to the lyrical songs. The volume or loudness of the track was also controlled by the participants themselves.

### Data collection

Depending on the categories, each song comprised of either a chorus or a verse, with the duration of approximately between 40 to 80 seconds. Each song was recorded three times. A 5-minutes break in between attempts was allowed, before they repeated the same steps to complete the four categories. In total, each participant spent around 45–75 minutes to finish the experiment.

MMG data from SCM and RA muscles were recorded throughout the singing sessions. The SCM and RA muscles’ activity were collected using BIOPAC® AcqKnowledge® acquisition software from MMG. The participants’ voice while singing was also recorded simultaneously using the Voice Memo application from iPhone 12 Mini.

### Data analysis

Muscle performance during quiet breathing and during singing (during inspiration and expiration) was monitored, recorded, and analyzed. In each round of singing, timing of breathe-in and breathe-out activities were identified from the voice recordings using Praat software (Praat, Paul Boersma and David Weenick, Phonetic Sciences Department, University of Amsterdam, The Netherlands).

The raw signal from BIOPAC® AcqKnowledge® acquisition software was sampled at 1 kHz frequency. The signal was then processed with a bandpass filter at lower and higher cut-off frequencies at 20 Hz and 200 Hz respectively. The bandpass filter was applied to reduce the additional noise that might have originated from motion artifacts. The amplitude of the signal was identified as voltages and retrieved as root mean square (RMS). All the processed data were exported for further analysis.

All data were analyzed using one-way analysis of variance (ANOVA) (SPSS Statistics, International Business Machines (IBM) Corporation, United States of America) to compare the accessory respiratory muscles activity while singing at different song categories. Least significant difference (LSD) test was conducted thereafter to determine any difference in accessory respiratory muscles activity while singing between specific categories. The significance level was fixed at 0.05.

## Results

After ANOVA analysis, MMG signal of the SCM muscle during quiet breathing, i.e. without singing, showed significant difference between inspiration and expiration activities (P < 0.05). However, MMG signal of the RA during quiet breathing showed no significant difference between inspiration and expiration activities.

The performance of SCM and RA muscles while singing were compared between different song categories using one-way ANOVA. Overall, there was a statistically significant difference among all categories (P < 0.01).

**Fig 3:**
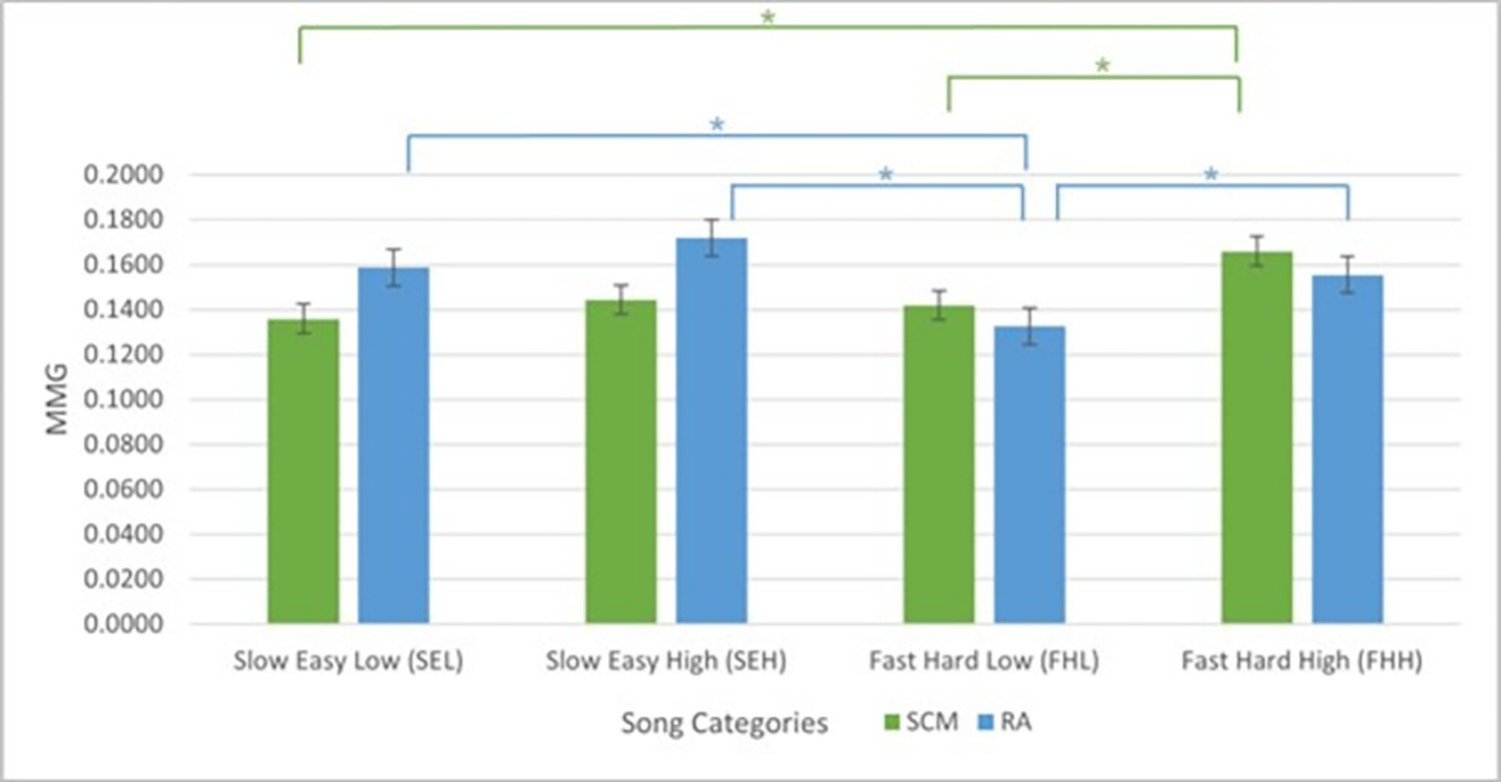
SCM and RA muscles performance while singing at different song categories. Note: * = P < 0.05.

In SCM, post-hoc least significant difference (LSD) test shows statistically significant difference between SEL and FHH (P = 0.008); and FHL and FHH categories (P = 0.035) (Table 2) (Fig 3). Meanwhile, in RA, LSD test showed statistically significant difference between FHL and FHH (P = 0.029) categories (Table 2) (Fig 3). There was also statistically significant difference between SEL and FHL (P = 0.013); and SEH and FHL (P < 0.001) categories in RA muscle (Table 2) (Fig 3).

**Table 2:** Post-hoc least significant difference (LSD) test results between song categories.

Muscle	Song category		Significant (p)
SCM	SEL	SEH	0.452
		FHL	0.594
		FHH	0.008
	SEH	FHL	0.827
		FHH	0.058
	FHL	FHH	0.035
RA	SEL	SEH	0.206
		FHL	0.013
		FHH	0.767
	SEH	FHL	<0.001
		FHH	0.118
	FHL	FHH	0.029

## Discussion

MMG signals from the SCM and RA muscles were always higher when singing compared to when not singing (P <0.05). This is in line with the role of accessory respiratory muscles like SCM and RA during deep breathing and heightened breathing demand activity such as singing. SCM performance during quiet breathing i.e., without singing reflects the nature of the accessory respiratory muscle during deep breathing [39, 40]. Meanwhile, RA was less active compared to SCM during quiet breathing. This is in parallel with the role of RA as accessory muscles during forceful expiration [41].

Music experts often labelled activation of the muscles of neck, shoulders, upper back, and chest as clavicular breathing [42]. This technique is often regarded as inefficient way of breathing for trained singers because it requires fatiguing way of elevating the rib cage while breathing in [42]. However, this could be beneficial in SCI, where main muscles of respiration might be affected after injury, including the diaphragm [43]. By activating accessory respiratory muscles like SCM, this may improve shortness of breath or dyspnoea in people with SCI [44, 45].

From Fig 3, it can be observed that the SCM muscle is most active when singing songs in high pitch. The SCI participants involuntarily activated their SCM muscle more during heightened breathing demands while singing. This outcome is consistent with many trained and classical singers, whereby their SCM muscles are most active at highest pitch [29–32]. This is important for people with SCI who have impaired diaphragm and intercostal muscles, especially those with cervical spinal cord injury.

In contrast to clavicular breathing, experts viewed abdominal breathing as the viable breathing mechanism for trained singers [46]. Abdominal breathing, or often referred as ‘belly breathing’ is when singers allow the full descent of the diaphragm by activating their abdominal muscles [46, 47]. By expanding the rib cage during inhalation, the intercostals allow the abdominal muscles to be active during expiratory [46, 47].

Fig 3 shows that the RA muscle is most active while singing songs with slow tempo and easy rhythmic complexity. Songs with easy rhythmic complexity have minimal note changes throughout the song, making it much easier for the participants to follow through. Combined with slow tempo, this creates more space and room for the SCI participants to inhale and exhale while singing, involuntarily utilising the ‘belly breathing’ technique.

Among classical singers, abdominal muscles were also more active throughout singing session compared to those of normal people [31]. Classical singers utilize ‘belly breathing’ that allows them to have more control on the speed which the chest collapses after inhalation. This technique is particularly useful for people with SCI level of T1-T11 to improve their trunk; whereby their diaphragm, intercostal, abdominal muscles are still intact.

In short, findings from this study have potential clinical application to clinical populations requiring breathing exercises or RMT for training and improving the strength of accessory muscles during singing therapy. Future research is needed to help clinicians in planning adequate singing therapy to strengthen the respiratory muscles in SCI, including investigation based on their levels of injury.

## Conclusion

This study has shown that different song characteristics (such as pitch, tempo, and rhythmic complexity) influenced the accessory respiratory muscles (such as SCM and RA) differently while singing. In high pitch songs, SCM muscle is more active compared to RA muscle. Meanwhile, RA muscle is more active while singing songs with easy tempo and rhythmic complexity. This could be beneficial for clinicians in prescribing singing therapy to improve the respiratory muscles impairment among people with SCI in the future. Further study is recommended to help clinicians estimate the intensity and duration of singing therapy needed to strengthen the respiratory muscles among people with SCI, including investigation based on their levels of injury.

## Supporting information

S1 Appendix Google Form filled by eight music experts in developing the song categories. (TIF)

S2 Appendix Characterization of the songs according to their pitch. (TIF)

S3 Appendix Characterization of the song into slow and fast tempo. (TIF)

S4 Appendix Song list. (TIF)
