# Supplementary figures and images for: Accessory respiratory muscles performance among people with spinal cord injury while singing songs with different musical parameters
Source: PLoS One. 2024 Jul 5;19(7):e0305940. doi: 10.1371/journal.pone.0305940 (PMC11226013; doi:10.1371/journal.pone.0305940)

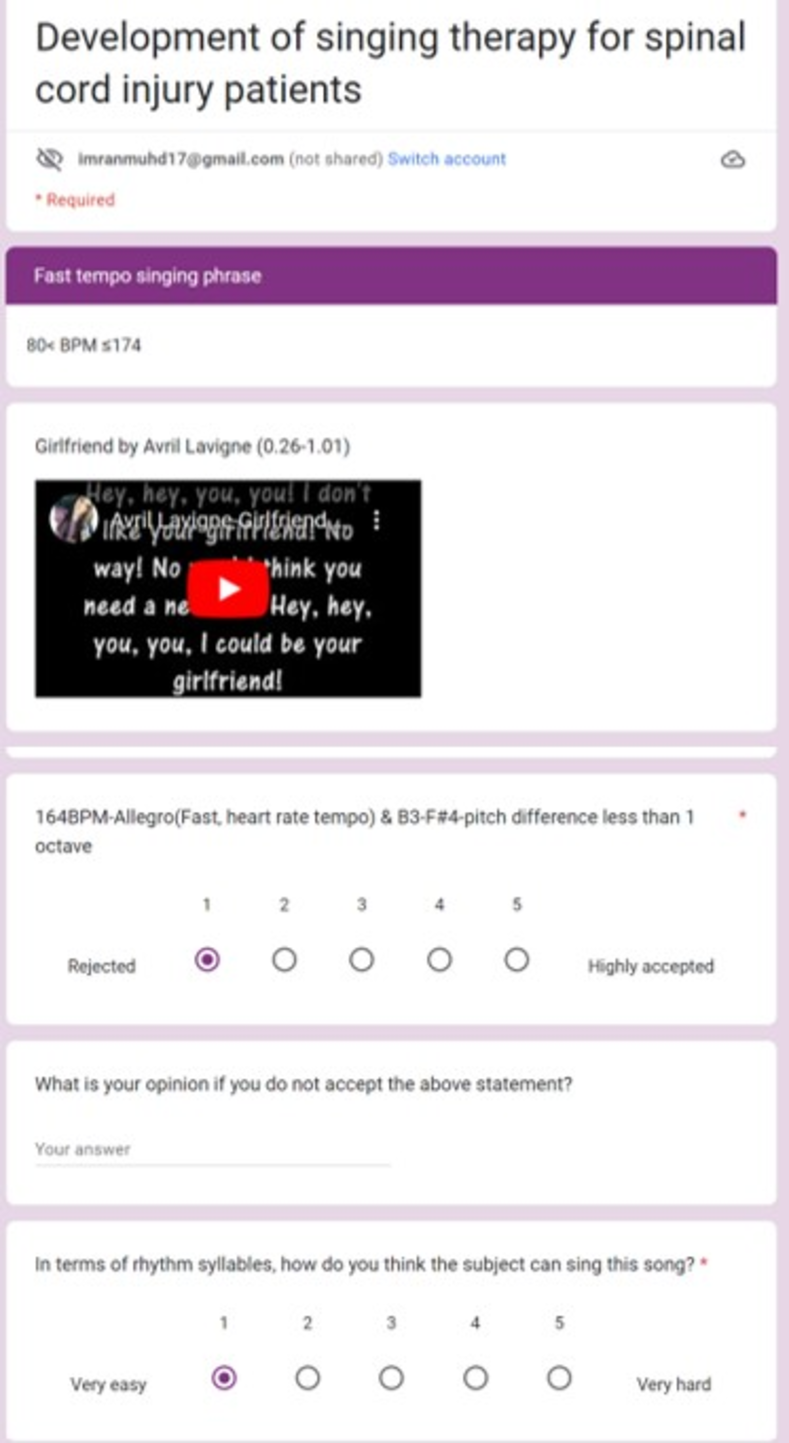

Supplement: S1 Appendix — (TIF) [file pone.0305940.s001.tif]

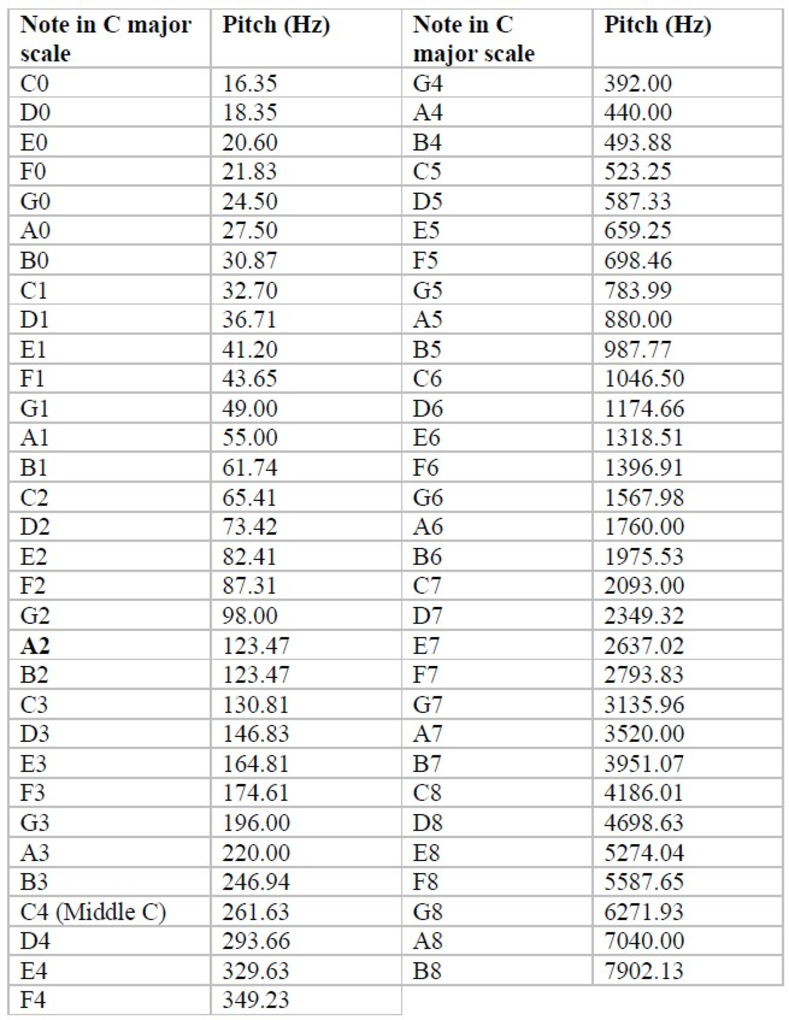

Supplement: S2 Appendix — (TIF) [file pone.0305940.s002.tif]

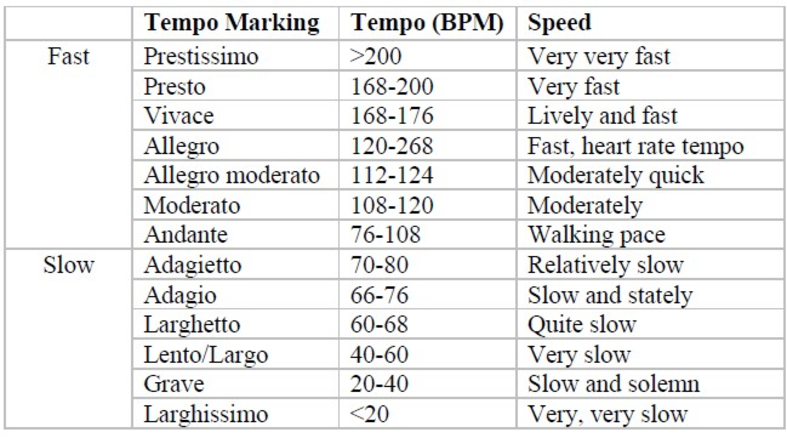

Supplement: S3 Appendix — (TIF) [file pone.0305940.s003.tif]

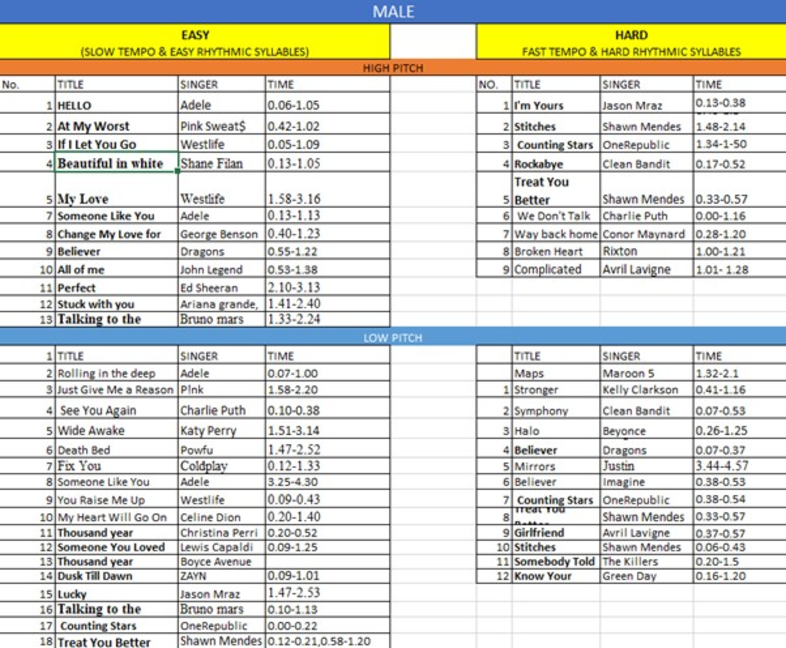

Supplement: S4 Appendix — (TIF) [file pone.0305940.s004.tif]
