# Supplementary material for: Accessory respiratory muscles performance among people with spinal cord injury while singing songs with different musical parameters
Source: PLoS One. 2024 Jul 5;19(7):e0305940. doi: 10.1371/journal.pone.0305940 (PMC11226013; doi:10.1371/journal.pone.0305940)
Supplement: Attachment — Submitted filename: Paper 3_PLOS One.docx [file pone.0305940.s005.docx]

**Accessory Respiratory Muscles Performance During Singing at Different Song Characteristics Among People with Spinal Cord Injury**

Muhammad Imran Ramli*^1^, Nur Azah Hamzaid^1,^, Julia Patrick Engkasan^2^, Juliana Usman^1^, Marzelan Salleh^3^, Wee Duen Hueh^1^

^1^Department of Biomedical Engineering, Faculty of Engineering, Universiti Malaya, 50603, Kuala Lumpur

^2^Department of Rehabilitation Medicine, Faculty of Medicine, Universiti Malaya, 50603, Kuala Lumpur

^3^Department of Music, Faculty of Creative Arts, Universiti Malaya, 50603, Kuala Lumpur

Corresponding author:

[imranramli@um.edu.my](mailto:azah.hamzaid@um.edu.my)

Department of Biomedical Engineering, Faculty of Engineering, University of Malaya, 50603, Kuala Lumpur

**ABSTRACT**

People with spinal cord injury (SCI) suffer from respiratory dysfunctions which include hypersecretions, bronchospasm and respiratory muscles weakness. Singing therapy has been implemented as part of respiratory muscle training (RMT) to improve their muscle strength. Singing different types and genres of songs may elicit specific recruitment of respiratory muscles, attributed to the variation of the songs characteristics including tempo, pitch, and rhythmic syllable. This study aims to determine the effect of singing songs with different characteristics on the accessory respiratory muscle performance among people with SCI. Thirteen male SCI participants were recruited. Respiratory muscle signals were retrieved by placing two mechanomyography (MMG) sensors on the sternocleidomastoid (SCM) and rectus abdominis (RA) muscles. Eight music experts categorized several songs into four categories based on their pitch, tempo, and rhythmic syllables. Each participant sang one song from each category. Findings showed statistically significant difference in RA and SCM responses among all categories (P < 0.01). The SCM muscle is most active while singing high pitch songs. Meanwhile, the RA is most active during slow tempo and easy rhythmic syllables. This shows that different accessory respiratory muscle is activated by people with SCI while singing songs with different characteristics. Clinicians could benefit from this outcome while prescribing singing therapy or exercise among people with SCI in the future.

Keywords: Singing; Accessory respiratory muscles; Spinal cord injury; Mechanomyography; Respiratory muscle training

**INTRODUCTION**

During quiet breathing, the inspiratory muscles (diaphragm and external intercostals) expand the thoracic cavity causing air to fill the lungs. This is followed by passive recoil of the chest wall during expiration. However, during heightened physical activities, accessory respiratory muscles are activated to keep up with the higher oxygen demand. These muscles include sternocleidomastoid, the scalenus, latissimus dorsi, and the abdominal muscles.

People with spinal cord injury (SCI) often have symptoms of respiratory insufficiency, particularly those with injury involving the cervical and thoracic region [1], [2]. The higher the level of lesion, the more respiratory muscles are impaired causing significant respiratory impairments. After SCI, the motor pathways that innervate the muscles of respiration are disrupted, leading to muscle weakness, spasticity, and paralysis [3]. People with SCI also suffer from impairment of respiratory motor control [4], limiting the recovery [5], [6] and quality of life [7].

Currently, clinicians and researchers have adopted several exercises to improve the respiratory muscles strength among people with SCI. This exercise is called respiratory muscle training (RMT). This training includes exercises for inspiration, expiration or both, of a certain duration and intensity to improve the strength and endurance of the muscles of respiration [8]. RMT includes resistive load training [9], [10], [11], [12], pressure threshold loading training [13], [14], [15], [16], normocapnic hyperpnea training [17], [18], [19], [20], and singing training [21].

Utilising singing as a training strategy to improve muscle strength among people with SCI is relatively new among researchers and clinicians compared to other conventional exercises. Singing has been shown to improve the respiratory strength, endurance and quality of life in many populations, such as degenerative speech/voice disorder [22], [23], [24], [25], chronic obstructive pulmonary disease (COPD) [26], and cancer patients [27], [28]. Like any other RMT, singing requires repeated and extensive inhalation and exhalation, leading to improvement in respiratory function [8].

In classical and trained singers, studies have shown that different muscles of respiration were recruited at different song parameters [29], [30], [31], [32]. Other than pitch, tempo and rhythmic syllable also affect the breathing mechanism while singing. Pitch refers to the control of the fundamental frequency of voice. The higher the pitch, the higher the tone of voice. Tempo refers to the speed of song in beats per minute (BPM). The faster the tempo, the faster the speed of song. Meanwhile, rhythmic syllable is when putting syllables of a phrase on the value of notes. Rapid changes in the syllables of a song makes it harder for someone to follow through.

Currently there are limited studies implementing singing training among people with SCI. Tamplin et al. (2013) have established trends of improvements in speech intensity, respiratory function, and muscle strength after singing a familiar song [21]. However, no study has investigated the effect of singing different characteristics of song among people with SCI. This knowledge is useful for clinical application with regards to selection of appropriate songs for RMT. Therefore, the study aims to determine the effect of singing at different tempo, pitch and rhythmic syllable on the accessory respiratory muscle activity while singing among people with SCI.

**MATERIALS AND METHODS**

In this study, MMG (BIOPAC MP150 system with 2-channel vibromyography) data was monitored and measured from their respective respiratory muscles while singing. These muscles include the sternocleidomastoid (SCM) and rectus abdominis (RA) muscles.

**Subjects**

A total of 13 individuals with SCI (13 males) were recruited in this study (Table 1). All participants were recruited between 3^rd^ June 2022 until 10^th^ August 2022. The authors had access to information that could identify individual participants during or after data collection. The authors obtained written consent from each participant before the experiment began. The inclusion criteria include SCI level between C4 and T11; American Spinal Injury Association Impairment Scale (AIS) A or B; aged 18 years and above. People with SCI above C4 level were not included to ensure all included participants’ diaphragm innervation is still intact. Meanwhile, any volunteers with injury level below T11 were not chosen because there is minimal respiratory muscle impairments. AIS A or B indicates the completeness of neurological deficits; A indicates complete motor and sensory injury whilst B indicates incomplete sensory injury. The exclusion criteria include any other disorders that could affect respiratory functions such as heart failure, coronavirus disease (COVID-19), and tuberculosis. Any volunteers who were unable to sing due to vocal cords conditions or sore throat were also excluded from this study.

**Study protocol**

The data was collected with SCI participants in a lab in the Department of Biomedical Engineering, Universiti Malaya, Kuala Lumpur. In addition, some data were also monitored and recorded in a non-governmental organization (NGO) building named Beautiful Gate Foundation for the Disabled in Kepong, Kuala Lumpur. The University of Malaya Research Ethics Committee (Ref: UM.TNC2/UMREC – 638) has approved the research ethics clearance before the protocol was conducted. Each participant provided informed consent prior to joining the experiment.

Researchers have collectively established MMG as a tool to demonstrate skeletal muscle performance through vibration in various clinical settings [33], [34], [35]. Studies using alternative devices in measuring the mechanical activities of the respiratory muscles such as MMG is imperative to complement electromyography (EMG) assessments [33]. Some researchers also suggested that a signal response that is inherently mechanical would be better represents the contraction of the muscle which led by the mechanical nature of the muscle fibre itself [36].

Two MMG sensors were attached to their SCM and RA muscles to observe the accessory respiratory muscles activity while singing. Both muscles were chosen because they are both superficial, which makes them palpable thus able to be located and captured by MMG. The MMG sensor on SCM were placed based on palpation of the muscle belly during manually resisted neck flexion contractions [37]. The sensor on RA was placed 2 cm lateral to the umbilicus, over the muscle mass [38].

**Song categorization**

In this study, 4 songs were chosen by the SCI participants from 4 different categories, in terms of their tempo, pitch and rhythmic syllable (Figure 1). A phrase with consistent tempo, pitch and rhythmic syllable for each song were identified (Figure 2). Eight music experts from the Department of Music, Faculty of Creative Arts, Universiti Malaya were recruited to evaluate the characteristics of the songs (Appendix A and D). Before singing, MMG signal during inspiration and expiration without singing was recorded.

All participants were required to choose one song from each category: (i) slow tempo easy low pitch (SEL), (ii) slow tempo easy high pitch (SEH), (iii) fast tempo hard low pitch (FHL) and (iv) fast tempo hard high pitch (FHH) (Figure 2). ‘Slow’ and ‘fast’ refer to the tempo of the song (Appendix C). ‘Easy’ and ‘hard’ refer to the rhythmic syllables. Meanwhile, ‘low’ and ‘high’ refer to the pitch of the song (Appendix B).

There were no specific instructions on how they were supposed to sing the song, except to follow the rhythm from the pre-recorded track. Every participant used headphones while listening to and singing to the lyrical songs. The volume or loudness of the track was also controlled by the participants themselves.

**Data collection**

Depending on the categories, each song comprised of either a chorus or a verse, with the duration approximately between 40 to 80 seconds. Each song was recorded under three different trials. A 5-minutes break in between each trial was allowed, before they repeated the same steps to complete the four categories. In total, each participant spent around 45-75 minutes to finish the experiment.

MMG data from SCM and RA muscles were recorded throughout the singing sessions. The SCM and RA muscles’ activity were collected using BIOPAC® AcqKnowledge® acquisition software from MMG. The participants’ voice while singing was also recorded simultaneously using the Voice Memo application from iPhone 12 Mini.

**Data analysis**

Muscle performance during quiet breathing and during singing (during inspiration and expiration) was monitored, recorded, and analysed. In each round of singing, timing of breathe-in and breathe-out activities were identified by referencing the voice recorded using Praat software (Praat, Paul Boersma and David Weenick, Phonetic Sciences Department, University of Amsterdam, The Netherlands). These timings were selected at the same section of the track for each participant.

The raw signal from BIOPAC® AcqKnowledge® acquisition software was sampled at 1 kHz frequency. The signal was then processed with a bandpass filter at lower and higher cut-off frequencies at 20 Hz and 200 Hz respectively. The bandpass filter was applied to reduce the additional noise that might have originated from motion artifacts. The amplitude of the signal was identified as voltages and retrieved as root mean square (RMS). All the processed data were exported for further analysis.

All data were analysed using one-way analysis of variance (ANOVA) (SPSS Statistics, International Business Machines (IBM) Corporation, United States of America) to compare the accessory respiratory muscles activity while singing at different song categories. Least significant difference (LSD) test was conducted thereafter to determine any difference in accessory respiratory muscles activity while singing between specific categories. The significance level was fixed at 0.05.

**RESULTS**

After ANOVA analysis, MMG signal of the SCM muscle during quiet breathing, i.e. without singing, showed significant difference between inspiration and expiration activities (P < 0.05). However, MMG signal of the RA during quiet breathing showed no significant difference between inspiration and expiration activities.

The performance of SCM and RA muscles while singings were compared between different song categories using one-way ANOVA. Overall, there was a statistically significant difference among all categories (P < 0.01).

In SCM, post-hoc least significant difference (LSD) test shows statistically significant difference between SEL and FHH (P < 0.01); and FHL and FHH categories (P < 0.05) (Figure 3). Meanwhile, in RA, LSD test shows statistically significant difference between FHL and FHH (P < 0.03) categories (Figure 3). There was also statistically significant difference between SEL and FHL (P < 0.02); and SEH and FHL (P < 0.001) categories in RA muscle (Figure 3).

**DISCUSSION**

MMG signal on the muscles while not singing is always lower compared to that of while singing (P <0.05). This is in line with the role of accessory respiratory muscles like SCM and RA during deep breathing and heightened breathing demand activity such as singing. SCM performance while quiet breathing i.e., without singing reflects the nature of the accessory respiratory muscle during deep breathing [39], [40]. Meanwhile, RA was less active compared to SCM during quiet breathing. This is in parallel with the role of RA as accessory muscles during forceful expiration [41].

Music experts often labelled activation on the muscles of neck, shoulders, upper back, and chest as clavicular breathing [42]. This technique is often regarded as inefficient way of breathing for trained singers because it requires fatiguing way of elevating the rib cage while breathing in [42]. However, this could be beneficial in SCI, where main muscles of respiration might be affected after injury, including the diaphragm [43]. By activating accessory respiratory muscles like SCM, this may improve their shortness of breath or dyspnoea after injury [44], [45].

From Figure 3, in can be observed that the SCM muscle is most active while singing songs in high pitch. The SCI participants involuntarily activated their SCM muscle more during heightened breathing demands while singing. This outcome is consistent in many trained and classical singers, whereby their SCM muscles are most active at highest pitch [29], [30], [31], [32]. This is important for people with SCI who have impaired diaphragm and intercostal muscles, especially those with cervical spinal cord injury.

In contrast to clavicular breathing, experts viewed abdominal breathing as the viable breathing mechanism for trained singers [46]. Abdominal breathing, or often referred as ‘belly breathing’ is when singers allow the full descent of the diaphragm by activating their abdominal muscles [46], [47]. By expanding the rib cage during inhalation, the intercostals allow the abdominal muscles to be active during expiratory [46], [47].

Figure 3 shows that the RA muscle is most active while singing songs with slow tempo and easy rhythmic syllables. Songs with easy rhythmic syllables have minimal note changes throughout the song, making it much easier for the participants to follow through. Combined with slow tempo, this creates more space and room for the SCI participants to inhale and exhale while singing, involuntarily utilising the ‘belly breathing’ technique.

Among classical singers, abdominal muscles were also more active throughout singing session compared to those of normal, trained students [31]. Classical singers utilise ‘belly breathing’ that allows them to have more control on the speed which the chest collapses after inhalation. This technique is particularly useful for people with SCI level of T1-T11 to improve their trunk; whereby their diaphragm, intercostal, abdominal muscles are still intact.

In short, findings from this study have potential clinical application to clinical populations requiring breathing exercises or RMT for training and improving the strength of accessory muscles during singing therapy. Future research is needed to help clinicians in planning adequate singing therapy to strengthen the respiratory muscles in SCI.

**CONCLUSION**

To conclude, this study has shown that different song characteristics (such as pitch, tempo, and rhythmic syllable) influenced the accessory respiratory muscles (such as SCM and RA) differently while singing. In high pitch songs, SCM muscle is more active compared to RA muscle. Meanwhile, RA muscle is more active while singing songs with easy tempo and rhythmic syllable. This could be beneficial for clinicians in prescribing singing therapy to improve the respiratory muscles impairment among people with SCI in the future. Further study is recommended to help clinicians estimate the intensity and duration of singing therapy needed to strengthen the respiratory muscles among people with SCI as an intervention measure.

**CONFLICT OF INTEREST**

The authors declare that the research was conducted in the absence of any commercial or financial relationships that could be construed as a potential conflict of interest.

**AUTHOR CONTRIBUTIONS**

NAH and JPE provided ideas for the research. MIR planned the methods to generate the results. NAH, JPE, JU and MS provided oversight and supervision, responsible for organization and implementation, and writing of the manuscript. MIR conducted the data collection/processing. MS and WDH helped in the making of the methodology. MIR was responsible for reporting data, including statistical analyses, evaluation, and presentation of the results. MIR wrote a substantive part of the manuscript. NAH, JPE and JU made critical review and revised manuscript for intellectual content. All authors read and approved the manuscript.

**FUNDING**

The first author, Muhammad Imran Ramli, received scholarship from *Skim Latihan Akademik Bumiputera* under Ministry of Higher Education Malaysia.

**ACKNOWLEDGEMENT**

The authors thank all the study participants for their time volunteering. The ethical approval was done by the University of Malaya Research Ethics Committee (Ref: UM.TNC2/UMREC - 638).

**REFERENCES**

[1] Baydur, A., Adkins, R. H., & Milic-Emili, J. (2001). Lung mechanics in individuals with spinal cord injury: effects of injury level and posture. *Journal of Applied Physiology (Bethesda, Md. : 1985)*, *90*(2), 405–411. <https://doi.org/10.1152/jappl.2001.90.2.405>

[2] van Houtte, S., Vanlandewijck, Y., & Gosselink, R. (2006). Respiratory muscle training in persons with spinal cord injury: a systematic review. *Respiratory Medicine*, *100*(11), 1886–1895. <https://doi.org/10.1016/j.rmed.2006.02.029>

[3] de Paleville, D. G. L. T., McKay, W. B., Folz, R. J., & Ovechkin, A. v. (2011). Respiratory Motor Control Disrupted by Spinal Cord Injury: Mechanisms, Evaluation, and Restoration. *Translational Stroke Research*, *2*(4), 463–473. <https://doi.org/10.1007/s12975-011-0114-0>

[4] Schilero, G. J., Spungen, A. M., Bauman, W. A., Radulovic, M., & Lesser, M. (2009). Pulmonary function and spinal cord injury. *Respiratory Physiology & Neurobiology*, *166*(3), 129–141. <https://doi.org/10.1016/j.resp.2009.04.002>

[5] Laffont, I., Durand, M.-C., Rech, C., de La Sotta, A. P., Hart, N., Dizien, O., & Lofaso, F. (2003). Breathlessness associated with abdominal spastic contraction in a patient with C4 tetraplegia: a case report. *Archives of Physical Medicine and Rehabilitation*, *84*(6), 906–908. <https://doi.org/10.1016/s0003-9993(02)04898-0>

[6] Linn, W. S., Adkins, R. H., Gong, H., & Waters, R. L. (2000). Pulmonary function in chronic spinal cord injury: a cross-sectional survey of 222 southern California adult outpatients. *Archives of Physical Medicine and Rehabilitation*, *81*(6), 757–763. <https://doi.org/10.1016/s0003-9993(00)90107-2>

[7] Walter, J. S., Sacks, J., Othman, R., Rankin, A. Z., Nemchausky, B., Chintam, R., & Wheeler, J. S. (2002). A database of self-reported secondary medical problems among VA spinal cord injury patients: its role in clinical care and management. *Journal of Rehabilitation Research and Development*, *39*(1), 53–61.

[8] Berlowitz, D. J., & Tamplin, J. (2013). Respiratory muscle training for cervical spinal cord injury ( Review ). *Cochrane Database of Systematic Reviews*, *23*(7), CD008507. <https://doi.org/10.1002/14651858.CD008507.pub2.www.cochranelibrary.com>

[9] Liaw, M. Y., Lin, M. C., Cheng, P. T., Wong, M. K. A., & Tang, F. T. (2000). Resistive inspiratory muscle training: Its effectiveness in patients with acute complete cervical cord injury. *Archives of Physical Medicine and Rehabilitation*, *81*(6), 752–756. <https://doi.org/10.1053/apmr.2000.5619>

[10] Litchke, L., Lloyd, L., Schmidt, E., Russian, C., & Reardon, R. (2010). Comparison of Two Concurrent Respiratory Resistance Devices on Pulmonary Function and Time Trial Performance of Wheel Chair Athletes. *Therapeutic Recreation Journal*, *44*(1), 51–62.

[11] Litchke, L. G., Russian, C. J., Lloyd, L. K., Schmidt, E. A., Price, L., & Walker, J. L. (2008). Effects of respiratory resistance training with a concurrent flow device on wheelchair athletes. *Journal of Spinal Cord Medicine*, *31*(1), 65–71. <https://doi.org/10.1080/10790268.2008.11753983>

[12] Roth, E. J., Stenson, K. W., Powley, S., Oken, J., Primack, S., Nussbaum, S. B., & Berkowitz, M. (2010). Expiratory Muscle Training in Spinal Cord Injury: A Randomized Controlled Trial. *Archives of Physical Medicine and Rehabilitation*, *91*(6), 857–861. <https://doi.org/10.1016/j.apmr.2010.02.012>

[13] Boswell-Ruys, C. L., Lewis, C. R. H., Wijeysuriya, N. S., McBaAin, R. A., Lee, B. B., McKenzie, D. K., Gandevia, S. C., & Butler, J. E. (2020). Impact of respiratory muscle training on respiratory muscle strength, respiratory function and quality of life in individuals with tetraplegia: A randomised clinical trial. *Thorax*, *75*(3), 279–288. <https://doi.org/10.1136/thoraxjnl-2019-213917>

[14] Postma, K., Haisma, J. A., Hopman, M. T. E., Bergen, M. P., Stam, H. J., & Bussmann, J. B. (2014). Resistive inspiratory muscle training in people with spinal cord injury during inpatient rehabilitation: A randomized controlled trial. *Physical Therapy*, *94*(12), 1709–1719. <https://doi.org/10.2522/ptj.20140079>

[15] Soumyashree, S., & Kaur, J. (2020). Effect of inspiratory muscle training (IMT) on aerobic capacity, respiratory muscle strength and rate of perceived exertion in paraplegics. *Journal of Spinal Cord Medicine*, *43*(1), 53–59. <https://doi.org/10.1080/10790268.2018.1462618>

[16] West, C. R., Taylor, B. J., Campbell, I. G., & Romer, L. M. (2014). Effects of inspiratory muscle training on exercise responses in Paralympic athletes with cervical spinal cord injury. *Scandinavian Journal of Medicine and Science in Sports*, *24*(5), 764–772. <https://doi.org/10.1111/sms.12070>

[17] Fischer, G., Tarperi, C., George, K., & Ardigò, L. P. (2014). An exploratory study of respiratory muscle endurance training in high lesion level paraplegic handbike athletes. *Clinical Journal of Sport Medicine*, *24*(1), 69–75. <https://doi.org/10.1097/JSM.0000000000000003>

[18] Mueller, G., Perret, C., & Hopman, M. T. E. (2008). Effects of respiratory muscle endurance training on wheelchair racing performance in athletes with paraplegia: A pilot study. *Clinical Journal of Sport Medicine*, *18*(1), 85–88. <https://doi.org/10.1097/JSM.0b013e318160c075>

[19] van Houtte, S., Vanlandewijck, Y., Kiekens, C., Spengler, C. M., & Gosselink, R. (2008). Patients with acute spinal cord injury benefit from normocapnic hyperpnoea training. *Journal of Rehabilitation Medicine*, *40*(2), 119–125. <https://doi.org/10.2340/16501977-0140>

[20] Xi, J., Jiang, H., Wang, J., Zhang, B., Cao, H., Yang, B., Frerichs, I., Möller, K., & Zhao, Z. (2019). Respiratory muscle endurance training with normocapnic hyperpnoea for patients with chronic spinal cord injury: A pilot short-term randomized controlled trial. *Journal of Rehabilitation Medicine*, *51*(8), 616–620. <https://doi.org/10.2340/16501977-2572>

[21] Tamplin, J., Baker, F. A., Grocke, D., Brazzale, D. J., Pretto, J. J., Ruehland, W. R., Buttifant, M., Brown, D. J., & Berlowitz, D. J. (2013). Effect of singing on respiratory function, voice, and mood after quadriplegia: A randomized controlled trial. *Archives of Physical Medicine and Rehabilitation*, *94*(3), 426–434. <https://doi.org/10.1016/j.apmr.2012.10.006>

[22] Azekawa, M., & Lagasse, A. B. (2018). Singing Exercises for Speech and Vocal Abilities in Individuals with Hypokinetic Dysarthria: A Feasibility Study. *Music Therapy Perspectives*, *36*(1), 40–49. <https://doi.org/10.1093/mtp/miw042>

[23] di Benedetto, P., Cavazzon, M., Mondolo, F., Rugiu, G., Peratoner, A., & Biasutti, E. (2009). Voice and choral singing treatment: a new approach for speech and voice disorders in Parkinson’s disease. *European Journal of Physical and Rehabilitation Medicine*, *45*(1), 13–19.

[24] Shih, L. C., Piel, J., Warren, A., Kraics, L., Silver, A., Vanderhorst, V., Simon, D. K., & Tarsy, D. (2012). Singing in groups for Parkinson’s disease (SING-PD): A pilot study of group singing therapy for PD-related voice/speech disorders. *Parkinsonism and Related Disorders*, *18*(5), 548–552. <https://doi.org/10.1016/j.parkreldis.2012.02.009>

[25] Stegemöller, E. L., Hibbing, P., Radig, H., & Wingate, J. (2017). Therapeutic singing as an early intervention for swallowing in persons with Parkinson’s disease. *Complementary Therapies in Medicine*, *31*, 127–133. <https://doi.org/10.1016/j.ctim.2017.03.002>

[26] Mcnamara, R. J., Epsley, C., Coren, E., & Mckeough, Z. J. (2017). Singing for adults with chronic obstructive pulmonary disease (COPD). In *Cochrane Database of Systematic Reviews*. <https://doi.org/10.1002/14651858.CD012296.pub2>

[27] Clements-Cortés, A. (2017). Singing and Vocal Interventions in Palliative and Cancer Care: Music Therapists’ Perceptions of Usage. *Journal of Music Therapy*, *54*(3), 336–361. <https://doi.org/10.1093/jmt/thx010>

[28] Fancourt, D., Williamon, A., Carvalho, L. A., Steptoe, A., Dow, R., & Lewis, I. (2016). Singing modulates mood, stress, cortisol, cytokine and neuropeptide activity in cancer patients and carers. *Ecancermedicalscience*, *10*, 1–13. <https://doi.org/10.3332/ecancer.2016.631>

[29] Pettersen, V. (2005). Muscular patterns and activation levels of auxiliary breathing muscles and thorax movement in classical singing. In *Folia Phoniatrica et Logopaedica* (Vol. 57, Issues 5–6, pp. 255–277). <https://doi.org/10.1159/000087079>

[30] Pettersen, V., Bjørkøy, K., Torp, H., & Westgaard, R. H. (2005). Neck and shoulder muscle activity and thorax movement in singing and speaking tasks with variation in vocal loudness and pitch. *Journal of Voice*, *19*(4), 623–634. <https://doi.org/10.1016/j.jvoice.2004.08.007>

[31] Pettersen, V., & Westgaard, R. H. (2004). Muscle activity in professional classical singing: A study on muscles in the shoulder, neck and trunk. *Logopedics Phoniatrics Vocology*, *29*(2), 56–65. <https://doi.org/10.1080/14015430410031661>

[32] Pettersen, V., & Westgaard, R. H. (2005). The activity patterns of neck muscles in professional classical singing. *Journal of Voice*, *19*(2), 238–251. <https://doi.org/10.1016/j.jvoice.2004.02.006>

[33] Ibitoye, M. O., Hamzaid, N. A., Zuniga, J. M., Hasnan, N., & Wahab, A. K. A. (2014). Mechanomyographic parameter extraction methods: An appraisal for clinical applications. *Sensors (Switzerland)*, *14*(12), 22940–22970. <https://doi.org/10.3390/s141222940>

[34] Islam, A., Sundaraj, K., Ahmad, R. B., & Ahamed, N. U. (2013). Mechanomyogram for Muscle Function Assessment : A Review. *PloS ONE*, *8*(3). <https://doi.org/10.1371/journal.pone.0058902>

[35] Ramli, M. I., Hamzaid, N. A., & Engkasan, J. P. (2019). Monitoring Breathing Muscle Performance During Singing Noninvasively Using Mechanomyography and Electromyography. *Journal of Voice*. <https://doi.org/10.1016/j.jvoice.2019.06.006>

[36] Gerdle, B., Karlsson, S., Day, S., & Djupsjöbacka, M. (1999). Acquisition, Processing and Analysis of the Surface Electromyogram. In *Modern Techniques in Neuroscience Research*. <https://doi.org/10.1007/978-3-642-58552-4_26>

[37] Falla, D., Dall’Alba, P., Rainoldi, A., Merletti, R., & Jull, G. (2002). Repeatability of surface EMG variables in the sternocleidomastoid and anterior scalene muscles. *European Journal of Applied Physiology*, *87*(6), 542–549. <https://doi.org/10.1007/s00421-002-0661-x>

[38] Marshall, P., & Murphy, B. (2003). The validity and reliability of surface EMG to assess the neuromuscular response of the abdominal muscles to rapid limb movement. *Journal of Electromyography and Kinesiology*, *13*(5), 477–489. <https://doi.org/10.1016/S1050-6411(03)00027-0>

[39] Short, D. J., Silver, J. R., & Lehr, R. P. (1991). Electromyographic study of sternocleidomastoid and scalene muscles in tetraplegic subjects during respiration. *Disability and Rehabilitation*, *13*(2), 46–49. <https://doi.org/10.3109/03790799109166683>

[40] Vitti, M., Fujiwara, M., & Basmajian, J. v. (1973). The Integrated Roles of Longus Colli and Sternocleidomastoid Muscles : An Electromyographic Study ’. *Anat. Rec.*, *177*, 471–484.

[41] Ito, K., Nonaka, K., Ogaya, S., Ogi, A., Matsunaka, C., & Horie, J. (2016). Surface electromyography activity of the rectus abdominis, internal oblique, and external oblique muscles during forced expiration in healthy adults. *Journal of Electromyography and Kinesiology*, *28*, 76–81. <https://doi.org/10.1016/j.jelekin.2016.03.007>

[42] Rootberg, R. (2003). Clavicular Breathing, Held Shoulders and Related Issues: A Round Table Discussion. *Voice and Speech Review*, *3*(1), 278–295. <https://doi.org/10.1080/23268263.2003.10739415>

[43] Warren, P. M., Askew, G. N., Kissane, R. W. P., Egginton, S., & Kwok, J. C. F. (2020). Oxygen transport kinetics underpin rapid and robust diaphragm recovery following chronic spinal cord injury. *Journal of Physiology-London*, *0*, 1–26. <https://doi.org/10.1113/JP280684>

[44] Cardozo, C. P. (2007). Respiratory Complications of Spinal Cord Injury. *The Journal of Spinal Cord Medicine*, *30*(4), 307–308.

[45] Gee, C. M., Williams, A. M., West, C. R., Sheel, A. W., & Eves, N. D. (2019). Respiratory muscle training in athletes with cervical spinal cord injury : effects on cardiopulmonary function and exercise capacity. *Journal of Physiology*, *597*(14), 3673–3685. <https://doi.org/10.1113/JP277943>

[46] Salomoni, S., van den Hoorn, W., & Hodges, P. (2016). Breathing and singing: Objective characterization of breathing patterns in classical singers. *PLoS ONE*, *11*(5). <https://doi.org/10.1371/journal.pone.0155084>

[47] Watson, P. J., Hoit, J. D., Lansing, R. W., & Hixon, T. J. (1989). Abdominal Muscle Activity During Classical Singing. *Journal of Voice*, *3*(1), 24–31.

**Figures caption**

Figure 1: The process behind the categorization and characterization of the songs

Figure 2: The categories of song including examples. Note: RS = rhythmic syllable; BPM = beats per minute.

Fig 3: SCM and RA muscles performance while singing at different song categories. Note: * = P < 0.05.

**Table**

Table 1: Participant demographics

| Demographic | SCI participants (n=13) |
| --- | --- |
| Age (year) | 48.8 ± 8.6 |
| Height (m) | 1.6 ± 0.1 |
| Weight (kg) | 67.3 ± 11.6 |
| BMI (kg/m^3^) | 24.9 ± 4.0 |

Note: BMI = body mass index

**Appendices**

Appendix A: Google Form filled by eight music experts in developing the song categories.

Appendix B: Characterization of the songs according to their pitch.

| **Note in C major scale** | **Pitch (Hz)** | **Note in C major scale** | **Pitch (Hz)** |
| --- | --- | --- | --- |
| C0 | 16.35 | G4 | 392.00 |
| D0 | 18.35 | A4 | 440.00 |
| E0 | 20.60 | B4 | 493.88 |
| F0 | 21.83 | C5 | 523.25 |
| G0 | 24.50 | D5 | 587.33 |
| A0 | 27.50 | E5 | 659.25 |
| B0 | 30.87 | F5 | 698.46 |
| C1 | 32.70 | G5 | 783.99 |
| D1 | 36.71 | A5 | 880.00 |
| E1 | 41.20 | B5 | 987.77 |
| F1 | 43.65 | C6 | 1046.50 |
| G1 | 49.00 | D6 | 1174.66 |
| A1 | 55.00 | E6 | 1318.51 |
| B1 | 61.74 | F6 | 1396.91 |
| C2 | 65.41 | G6 | 1567.98 |
| D2 | 73.42 | A6 | 1760.00 |
| E2 | 82.41 | B6 | 1975.53 |
| F2 | 87.31 | C7 | 2093.00 |
| G2 | 98.00 | D7 | 2349.32 |
| **A2** | 123.47 | E7 | 2637.02 |
| B2 | 123.47 | F7 | 2793.83 |
| C3 | 130.81 | G7 | 3135.96 |
| D3 | 146.83 | A7 | 3520.00 |
| E3 | 164.81 | B7 | 3951.07 |
| F3 | 174.61 | C8 | 4186.01 |
| G3 | 196.00 | D8 | 4698.63 |
| A3 | 220.00 | E8 | 5274.04 |
| B3 | 246.94 | F8 | 5587.65 |
| C4 (Middle C) | 261.63 | G8 | 6271.93 |
| D4 | 293.66 | A8 | 7040.00 |
| E4 | 329.63 | B8 | 7902.13 |
| F4 | 349.23 |  |  |

Appendix C: Characterization of the song into slow and fast tempo.

|  | **Tempo Marking** | **Tempo (BPM)** | **Speed** |
| --- | --- | --- | --- |
| Fast | Prestissimo | >200 | Very very fast |
|  | Presto | 168-200 | Very fast |
|  | Vivace | 168-176 | Lively and fast |
|  | Allegro | 120-268 | Fast, heart rate tempo |
|  | Allegro moderato | 112-124 | Moderately quick |
|  | Moderato | 108-120 | Moderately |
|  | Andante | 76-108 | Walking pace |
| Slow | Adagietto | 70-80 | Relatively slow |
|  | Adagio | 66-76 | Slow and stately |
|  | Larghetto | 60-68 | Quite slow |
|  | Lento/Largo | 40-60 | Very slow |
|  | Grave | 20-40 | Slow and solemn |
|  | Larghissimo | <20 | Very, very slow |

Appendix D: Song list.
