# Supplementary material for: Accessory respiratory muscles performance among people with spinal cord injury while singing songs with different musical parameters
Source: PLoS One. 2024 Jul 5;19(7):e0305940. doi: 10.1371/journal.pone.0305940 (PMC11226013; doi:10.1371/journal.pone.0305940)
Supplement: Attachment — Submitted filename: PLOS One - reviewer comments.docx [file pone.0305940.s007.docx]

PLoS One reviewer comments:

This is an interesting and well written article describing the results of an exploratory study of accessory respiratory muscle activation for people with spinal cord injury during singing songs with different characteristics.

Overall, the paper is clearly laid out and all sections are articulated well. There are only a few minor things for consideration that I have outlined below.

Would muscle ‘activation’ be a better word choice than ‘performance’ in the title? Also, I’m not sure about the grammar of “singing at different song characteristics”. Maybe “songs with different musical parameters”?

Avoid use of pathologizing language such as “suffer from” (first line of abstract). More neutral or strengths-based language is preferred, such as “experience” or “live with”

I’m also not sure about phrase “rhythmic syllable” as one of the musical parameters of songs referred to throughout the paper. I think what you mean is “rhythmic complexity” or “syllable rhythmic complexity” if you want to keep the reference to syllables in there.

In the abstract it would be helpful to state the level of SCI. Eg. participants with SCI (*C4 -T11 ASIA A or B*).

Last line of abstract, suggest replacing “outcome” with “knowledge” – “Clinicians could benefit from this knowledge…”

Please state the exact number of songs that were characterized into the four categories.

Introduction is clear and succinct and supported by appropriate current references.

A clear summary of current published knowledge in the area and justification for the study.

Line 51-52 – suggest joining these 2 sentences for better flow: “*This exercise is called respiratory muscle training (RMT) and includes exercises for inspiration, expiration or both of a certain duration and intensity to improve the strength and endurance of the muscles of respiration.”*

Revise lines 65-66 based on comments above about the grammar of “singing at different song characteristics”. Maybe “songs with different musical parameters”? Also “rhythmic complexity” rather than “rhythmic syllable”

Revise lines 69-70 for clarity of expression. Perhaps “*rhythmic complexity refers to complexity of the rhythm of sung syllables. Rapid changes in the syllables of a song and complex or syncopated rhythm make it more difficult to sing.”*

Line 73 has a reference date which is not required in this referencing style.

Method and Results are clearly expressed. Appropriate use of tables and figures.

Line 82 – suggest removing the words “their respective respiratory muscles” and joining these two sentences.

Line 92 – suggest changing the word “chosen” to “eligible” and modifying this sentence:

“…*volunteers with injury level below T11 were not eligible because there is minimal respiratory muscle impairment below this level of injury.”*

Line 97 – typo – “vocal cord conditions”

Discussion and Implications are comprehensive and appropriate reflect on findings and study limitations in relation to previous research.

Possible typo on p12 line 359 “positive effects ***on*** other cognitive functions…”

Line 104 – remove the word “has”.

Lines 109-111

*“Studies using alternative devices in measuring the mechanical activities of the respiratory muscles such as MMG are imperative to complement electromyography (EMG) assessments [33]. Some researchers have also suggested that a signal response that is inherently mechanical…”*

Lines 114-115

*“Two MMG sensors were attached to participants’ SCM and RA muscles to observe ~~the~~ accessory respiratory muscle activity while singing. These two muscles were chosen…”*

Line 117

Plural error. Was there 1 or 2 MMG sensors place on SCM?

*“MMG sensor on SCM was placed” or “MMG sensors on SCM were placed”*

Line 127

*“All participants were required to choose one song from each category.”* Figure 2 shows only 1 song in each category though. Did they choose really?

Line 133
Suggest changing “follow the rhythm” to “follow the musical cues”

Line 138

What do you mean by: “*Each song was recorded under three different trials”?*

Line 144-145 What were the voice memo recordings used for?

Line 149 Suggest changing: “*breathe-in and breathe-out activities were identified by referencing the voice recorded using Praat software”* to “*breathe-in and breathe-out activities were identified from the voice recordings using Praat software”*

Line 152 *“These timings were selected at the same section of the track for each participant.”*

Didn’t each participant choose different songs though?

Results

Please add a results table and ensure to use exact p values in the results section (not just p<0.05). Past tense also required. (eg. Line 173 “(LSD) test showed…”

It would be interesting to know if there were any differences in accessory respiratory muscle activation for people with different injury levels.

Discussion

Please use past tense and don’t present results/stats in discussion.

Line 180 – “*MMG signal on the muscles while not singing is always lower compared to that of while singing (P <0.05).”* Suggested revision: “*MMG signals from the SCM and RA muscles were always higher when singing compared to when not singing.”*

Line 192 – please clarify who you mean by “their”. Ie. “*By activating accessory respiratory muscles like SCM, this may improve shortness of breath or dyspnoea for people with SCI.*

Line 207 – please clarify what you mean by *“follow through”.*

Line 210 – the meaning of this sentence is unclear. Who are the “*normal trained students*”? Are these singing students? If so, this is a confusing comparison with classical singers.

*“Among classical singers, abdominal muscles were also more active throughout singing session compared to those of normal, trained students.”*

Conclusion – please remove the unnecessary words “to conclude” in line 220

For Editor

I do not have any potential or perceived competing interests that may influence my review.

This is a very well written and clear constructed paper with interesting results.

Happy to recommend for publication with the above revisions!
