# Supplementary material for: Accessory respiratory muscles performance among people with spinal cord injury while singing songs with different musical parameters
Source: PLoS One. 2024 Jul 5;19(7):e0305940. doi: 10.1371/journal.pone.0305940 (PMC11226013; doi:10.1371/journal.pone.0305940)
Supplement: Attachment — Submitted filename: Response to Reviewers Imran PlosOne.docx [file pone.0305940.s008.docx]

A Point-by-Point Response to Reviewers’ Comments to the Author

| No. | Reviewer’s comment | Response |
| --- | --- | --- |
| 1. | Would muscle ‘activation’ be a better word choice than ‘performance’ in the title? | Thank you for your suggestion. However, in our case ‘performance’ is better as we were using mechanomyography (MMG), not electromyography (EMG).  EMG measures the electrical activity of the muscle during contraction generated by the brain. Meanwhile, MMG measures the mechanical vibrations produced by the muscle contraction. |
| 2. | Also, I’m not sure about the grammar of “singing at different song characteristics”. Maybe “songs with different musical parameters”? | I have rearranged the title of the manuscript according to your recommendation from “*Accessory* *Respiratory Muscles Performance During Singing at Different Song Characteristics Among People with Spinal Cord Injury*” into “**Accessory Respiratory Muscles Performance Among People with Spinal Cord Injury While Singing Songs with Different Musical Parameters**”. |
| 3. | Avoid use of pathologizing language such as “suffer from” (first line of abstract). More neutral or strengths-based language is preferred, such as “experience” or “live with”. | The sentence is changed from “suffer from” into “experience”. |
| 4. | I’m also not sure about the phrase “rhythmic syllable” as one of the musical parameters of songs referred to throughout the paper. I think what you mean is “rhythmic complexity” or “syllable rhythmic complexity” if you want to keep the reference to syllables in there. | Thank you for your input. The phrase “rhythmic syllable” is changed into “rhythmic complexity’” as pointed out by the reviewer, throughout the manuscript. |
| 5. | In the abstract it would be helpful to state the level of SCI. Eg. participants with SCI (*C4 -T11 ASIA A or B*). | We have included the participants ASIA classification and injury level in the abstract. Thank you for the suggestion. |
| 6. | Last line of abstract, suggest replacing “outcome” with “knowledge” – “Clinicians could benefit from this knowledge…” | The word “outcome” has been replaced with the word “knowledge” in the last line of abstract. |
| 7. | Please state the exact number of songs that were characterized into the four categories. | The exact number of songs is added into the manuscript in line 122. Also, the whole paragraph has been rearranged to ensure less confusion from readers. |
| 8. | Line 51-52 – suggest joining these 2 sentences for better flow: “*This exercise is called respiratory muscle training (RMT) and includes exercises for inspiration, expiration or both of a certain duration and intensity to improve the strength and endurance of the muscles of respiration.”* | The 2 sentences in line 51-52 have been combined as mentioned by the reviewer. |
| 9. | Revise lines 65-66 based on comments above about the grammar of “singing at different song characteristics”. Maybe “songs with different musical parameters”? Also “rhythmic complexity” rather than “rhythmic syllable” | Changes has been made accordingly in both cases. |
| 10. | Revise lines 69-70 for clarity of expression. Perhaps “*rhythmic complexity refers to complexity of the rhythm of sung syllables. Rapid changes in the syllables of a song and complex or syncopated rhythm make it more difficult to sing.”* | Thank you for the correction. The lines 69-70 has been revised as stated by the reviewer. |
| 11. | Line 73 has a reference date which is not required in this referencing style. | I have double checked the reference with ScienceDirect, and it still stated the same year as the one in this manuscript, which is 2013. |
| 12. | Line 82 – suggest removing the words “their respective respiratory muscles” and joining these two sentences. | Changes has been made accordingly. |
| 13. | Line 92 – suggest changing the word “chosen” to “eligible” and modifying this sentence:  “…*volunteers with injury level below T11 were not eligible because there is minimal respiratory muscle impairment below this level of injury.”* | Line 92 has been revised as stated by the reviewer. |
| 14. | Line 97 – typo – “vocal cord conditions” | Noted. The word “cords” has been changed into “cord”. |
| 15. | Line 104 – remove the word “has”. | The word “has” has been removed. |
| 16. | Lines 109-111  *“Studies using alternative devices in measuring the mechanical activities of the respiratory muscles such as MMG are imperative to complement electromyography (EMG) assessments [33]. Some researchers have also suggested that a signal response that is inherently mechanical…”* | Thank you for pointing out the grammar mistakes. Changes has been made accordingly. |
| 17. | Lines 114-115  *“Two MMG sensors were attached to participants’ SCM and RA muscles to observe ~~the~~ accessory respiratory muscle activity while singing. These two muscles were chosen…”* | Changes has been made accordingly. |
| 18. | Line 117  Plural error. Was there 1 or 2 MMG sensors place on SCM?  *“MMG sensor on SCM was placed” or “MMG sensors on SCM were placed”* | There was only one sensor placed on SCM. Therefore, the correct sentence would be “*MMG sensor on SCM was placed”.* Changes has been made on line 117. |
| 19. | Line 127  *“All participants were required to choose one song from each category.”* Figure 2 shows only 1 song in each category though. Did they choose really? | Participants were given a list of songs for each category, as shown in Appendix D. There are 48 songs on the list (as addressed in point no. 7).  From that list, the participants will choose only one song for each category.  Figure 2 only showed the four quadrants (or categories); (i) slow tempo easy low pitch (SEL), (ii) slow tempo easy high pitch (SEH), (iii) fast tempo hard low pitch (FHL) and (iv) fast tempo hard high pitch (FHH). |
| 20. | Line 133  Suggest changing “follow the rhythm” to “follow the musical cues” | Changes has been made as suggested. |
| 21. | Line 138  What do you mean by: “*Each song was recorded under three different trials”?* | Each song was recorded for three attempts. The sentence has been changed to make sure less confusion from the readers. |
| 22. | Line 144-145 What were the voice memo recordings used for? | The voice memo recordings were used together with Praat software to identify the exact moments of inspiration and expiration for data analysis. |
| 23. | Line 149 Suggest changing: “*breathe-in and breathe-out activities were identified by referencing the voice recorded using Praat software”* to “*breathe-in and breathe-out activities were identified from the voice recordings using Praat software”* | Thank you for the suggestion. Changes has been made as stated by the reviewer. |
| 24. | Line 152 *“These timings were selected at the same section of the track for each participant.”*  Didn’t each participant choose different songs though? | Yes, you are right. This is a mistake from my end. The sentence has been removed from the manuscript. |
| 25. | Please add a results table and ensure to use exact p values in the results section (not just p<0.05). Past tense also required. (eg. Line 173 “(LSD) test showed…” | A new table (Table 2) has been added on the manuscript. The exact p values were also stated in the result section. The grammar error on Line 173 has also been corrected. |
| 26. | It would be interesting to know if there were any differences in accessory respiratory muscle activation for people with different injury levels. | We did not perform any specific analysis to compare the accessory respiratory muscle activation between participants of different injury levels, thus are unable to provide a conclusive statement addressing this point.  However, based on our crude observation, we do not see any difference in muscle performance between the different injury levels.  We have added this in the discussion (line 217, page 10)  “Future research is needed to help clinicians in planning adequate singing therapy to strengthen the respiratory muscles in SCI, including investigation based on their levels of injury.” |
| 27. | Please use past tense and don’t present results/stats in discussion.  Line 180 – “*MMG signal on the muscles while not singing is always lower compared to that of while singing (P <0.05).”* Suggested revision: “*MMG signals from the SCM and RA muscles were always higher when singing compared to when not singing.”* | Changes has been made accordingly. |
| 28. | Line 192 – please clarify who you mean by “their”. Ie. “*By activating accessory respiratory muscles like SCM, this may improve shortness of breath or dyspnoea for people with SCI.* | Thank you for the suggestion. The sentence has been rearranged in the manuscript. |
| 29. | Line 207 – please clarify what you mean by *“follow through”.* | From our experience during data collection, some participants found it harder to sing all the lyrics in songs categorized as hard rhythmic complexity. Some participants could not keep up with the lyrics due to the fast nature of the songs. |
| 30. | Line 210 – the meaning of this sentence is unclear. Who are the “*normal trained students*”? Are these singing students? If so, this is a confusing comparison with classical singers.  *“Among classical singers, abdominal muscles were also more active throughout singing session compared to those of normal, trained students.”* | Thank you for notifying us with the mistake. Correction has been made accordingly. |
| 31. | please remove the unnecessary words “to conclude” in line 220 | The word “to conclude” has been removed. |
