# Supplementary material for: Accessory respiratory muscles performance among people with spinal cord injury while singing songs with different musical parameters
Source: PLoS One. 2024 Jul 5;19(7):e0305940. doi: 10.1371/journal.pone.0305940 (PMC11226013; doi:10.1371/journal.pone.0305940)
Supplement: Attachment — Submitted filename: Point by point responses.docx [file pone.0305940.s009.docx]

Point-by-point responses to the editor comments.

| No. | Reviewer’s comment | Response |
| --- | --- | --- |
| 1. | Please get your article reviewed from a language expert. | The manuscript has been proofread by a proofreader. A certificate from the proofreader is attached in the appendix below. |
| 2. | Please get your article reviewed from the technical language related to musicology must be simple and concise to enhance its readability. | The manuscript has been proofread by two experts from music background: (1) Dr Marzelan Salleh, one of the co-authors of the manuscript from Music Department in Faculty of Creative Arts in Universiti Malaya, and (2) Dr Camellia Siti Maya Mohamed Razali from Music Department in Faculty of Human Ecology in Universiti Putra Malaysia. |
| 3. | Please review your reference list to ensure that it is complete and correct. If you have cited papers that have been retracted, please include the rationale for doing so in the manuscript text or remove these references and replace them with relevant current references. | The reference list has been reviewed and several changes has been made in the DOI link. The changes were made because some of the DOI links were not working. This includes the link to references number 8, 9, 40, 41, 44, and 47. None of the references were retracted after reviewing the list. |
| 4. | While revising your submission, please upload your figure files to the Preflight Analysis and Conversion Engine (PACE) digital diagnostic tool, https://pacev2.apexcovantage.com/. PACE helps ensure that figures meet PLOS requirements. | All figures from the manuscript have been uploaded in PACE website. The updated and corrected version of the figures have been uploaded during submission. |

Appendix A: Certificate by the proofreader of the manuscript.
