# Supplementary material for: Accessory respiratory muscles performance among people with spinal cord injury while singing songs with different musical parameters
Source: PLoS One. 2024 Jul 5;19(7):e0305940. doi: 10.1371/journal.pone.0305940 (PMC11226013; doi:10.1371/journal.pone.0305940)
Supplement: Peer review history

# Peer review: Decision Letter 0

**DOI:** 10.1371/journal.pone.0305940.r001

**Authors:** Sahreen Anwar

25 Jul 2023

PONE-D-23-07503Accessory Respiratory Muscles Performance During Singing at Different Song Characteristics Among People with Spinal Cord InjuryPLOS ONE

Dear Dr. Ramli

Thank you for submitting your manuscript to PLOS ONE. After careful consideration, we feel that it has merit but does not fully meet PLOS ONE’s publication criteria as it currently stands. Therefore, we invite you to submit a revised version of the manuscript that addresses the points raised during the review process.

Please submit your revised manuscript by Sep 08 2023 11:59PM. If you will need more time than this to complete your revisions, please reply to this message or contact the journal office at plosone@plos.org. When you're ready to submit your revision, log on to https://www.editorialmanager.com/pone/ and select the 'Submissions Needing Revision' folder to locate your manuscript file.

Please include the following items when submitting your revised manuscript:

- A rebuttal letter that responds to each point raised by the academic editor and reviewer(s). You should upload this letter as a separate file labeled 'Response to Reviewers'.
- A marked-up copy of your manuscript that highlights changes made to the original version. You should upload this as a separate file labeled 'Revised Manuscript with Track Changes'.
- An unmarked version of your revised paper without tracked changes. You should upload this as a separate file labeled 'Manuscript'.

If you would like to make changes to your financial disclosure, please include your updated statement in your cover letter. Guidelines for resubmitting your figure files are available below the reviewer comments at the end of this letter.

If applicable, we recommend that you deposit your laboratory protocols in protocols.io to enhance the reproducibility of your results. Protocols.io assigns your protocol its own identifier (DOI) so that it can be cited independently in the future. For instructions see: https://journals.plos.org/plosone/s/submission-guidelines#loc-laboratory-protocols. Additionally, PLOS ONE offers an option for publishing peer-reviewed Lab Protocol articles, which describe protocols hosted on protocols.io. Read more information on sharing protocols at https://plos.org/protocols?utm_medium=editorial-email&utm_source=authorletters&utm_campaign=protocols.

We look forward to receiving your revised manuscript.

Kind regards,

Sahreen Anwar, PhD PT

Academic Editor

PLOS ONE

Journal Requirements:

When submitting your revision, we need you to address these additional requirements.

1. Please ensure that your manuscript meets PLOS ONE's style requirements, including those for file naming. The PLOS ONE style templates can be found at

https://journals.plos.org/plosone/s/file?id=wjVg/PLOSOne_formatting_sample_main_body.pdf and

https://journals.plos.org/plosone/s/file?id=ba62/PLOSOne_formatting_sample_title_authors_affiliations.pdf

2. We note that you have indicated that data from this study are available upon request. PLOS only allows data to be available upon request if there are legal or ethical restrictions on sharing data publicly. For more information on unacceptable data access restrictions, please see http://journals.plos.org/plosone/s/data-availability#loc-unacceptable-data-access-restrictions.

In your revised cover letter, please address the following prompts:

    a) If there are ethical or legal restrictions on sharing a de-identified data set, please explain them in detail (e.g., data contain potentially sensitive information, data are owned by a third-party organization, etc.) and who has imposed them (e.g., an ethics committee). Please also provide contact information for a data access committee, ethics committee, or other institutional body to which data requests may be sent.

   b) If there are no restrictions, please upload the minimal anonymized data set necessary to replicate your study findings as either Supporting Information files or to a stable, public repository and provide us with the relevant URLs, DOIs, or accession numbers. For a list of acceptable repositories, please see http://journals.plos.org/plosone/s/data-availability#loc-recommended-repositories.

We will update your Data Availability statement on your behalf to reflect the information you provide.

3. PLOS requires an ORCID iD for the corresponding author in Editorial Manager on papers submitted after December 6th, 2016. Please ensure that you have an ORCID iD and that it is validated in Editorial Manager. To do this, go to ‘Update my Information’ (in the upper left-hand corner of the main menu), and click on the Fetch/Validate link next to the ORCID field. This will take you to the ORCID site and allow you to create a new iD or authenticate a pre-existing iD in Editorial Manager. Please see the following video for instructions on linking an ORCID iD to your Editorial Manager account: https://www.youtube.com/watch?v=_xcclfuvtxQ

4. Please upload a new copy of Figures 1, 2 and 3 as the detail is not clear. Please follow the link for more information: " ext-link-type="uri" xlink:type="simple">https://blogs.plos.org/plos/2019/06/looking-good-tips-for-creating-your-plos-figures-graphics/" https://blogs.plos.org/plos/2019/06/looking-good-tips-for-creating-your-plos-figures-graphics/

5. Please review your reference list to ensure that it is complete and correct. If you have cited papers that have been retracted, please include the rationale for doing so in the manuscript text, or remove these references and replace them with relevant current references. Any changes to the reference list should be mentioned in the rebuttal letter that accompanies your revised manuscript. If you need to cite a retracted article, indicate the article’s retracted status in the References list and also include a citation and full reference for the retraction notice.

[Note: HTML markup is below. Please do not edit.]

Reviewers' comments:

Reviewer's Responses to Questions

**Comments to the Author**

1. Is the manuscript technically sound, and do the data support the conclusions?

The manuscript must describe a technically sound piece of scientific research with data that supports the conclusions. Experiments must have been conducted rigorously, with appropriate controls, replication, and sample sizes. The conclusions must be drawn appropriately based on the data presented. 

Reviewer #1: Yes

Reviewer #2: Yes

**********

2. Has the statistical analysis been performed appropriately and rigorously? 

Reviewer #1: Yes

Reviewer #2: I Don't Know

**********

3. Have the authors made all data underlying the findings in their manuscript fully available?

The PLOS Data policy requires authors to make all data underlying the findings described in their manuscript fully available without restriction, with rare exception (please refer to the Data Availability Statement in the manuscript PDF file). The data should be provided as part of the manuscript or its supporting information, or deposited to a public repository. For example, in addition to summary statistics, the data points behind means, medians and variance measures should be available. If there are restrictions on publicly sharing data—e.g. participant privacy or use of data from a third party—those must be specified.

Reviewer #1: No

Reviewer #2: No

**********

4. Is the manuscript presented in an intelligible fashion and written in standard English?

PLOS ONE does not copyedit accepted manuscripts, so the language in submitted articles must be clear, correct, and unambiguous. Any typographical or grammatical errors should be corrected at revision, so please note any specific errors here.

Reviewer #1: Yes

Reviewer #2: Yes

**********

5. Review Comments to the Author

Please use the space provided to explain your answers to the questions above. You may also include additional comments for the author, including concerns about dual publication, research ethics, or publication ethics. (Please upload your review as an attachment if it exceeds 20,000 characters)

Reviewer #1: I believe that this is the original work of the authors and presents a novel idea and appropriate methodology. the study is well planned and conducted accordingly. the selection of he songs and their characteristics is reasonable and the results may be utilized further in patients with SCI.

Reviewer #2: This is an interesting and well written article describing the results of an exploratory study of accessory respiratory muscle activation for people with spinal cord injury during singing songs with different characteristics.

Overall, the paper is clearly laid out and all sections are articulated well. There are only a few minor things for consideration that I have outlined below.

Would muscle ‘activation’ be a better word choice than ‘performance’ in the title? Also, I’m not sure about the grammar of “singing at different song characteristics”. Maybe “songs with different musical parameters”?

Avoid use of pathologizing language such as “suffer from” (first line of abstract). More neutral or strengths-based language is preferred, such as “experience” or “live with”

I’m also not sure about phrase “rhythmic syllable” as one of the musical parameters of songs referred to throughout the paper. I think what you mean is “rhythmic complexity” or “syllable rhythmic complexity” if you want to keep the reference to syllables in there.

In the abstract it would be helpful to state the level of SCI. Eg. participants with SCI (C4 -T11 ASIA A or B).

Last line of abstract, suggest replacing “outcome” with “knowledge” – “Clinicians could benefit from this knowledge…”

Please state the exact number of songs that were characterized into the four categories.

Introduction is clear and succinct and supported by appropriate current references.

A clear summary of current published knowledge in the area and justification for the study.

Line 51-52 – suggest joining these 2 sentences for better flow: “This exercise is called respiratory muscle training (RMT) and includes exercises for inspiration, expiration or both of a certain duration and intensity to improve the strength and endurance of the muscles of respiration.”

Revise lines 65-66 based on comments above about the grammar of “singing at different song characteristics”. Maybe “songs with different musical parameters”? Also “rhythmic complexity” rather than “rhythmic syllable”

Revise lines 69-70 for clarity of expression. Perhaps “rhythmic complexity refers to complexity of the rhythm of sung syllables. Rapid changes in the syllables of a song and complex or syncopated rhythm make it more difficult to sing.”

Line 73 has a reference date which is not required in this referencing style. NB. Tamplin et all have a 2011 study which is more aligned with the exploratory design of this study and should be referenced here and compared in the discussion also.

Tamplin, J., Brazzale, D., Pretto, J.J., Ruehland, W.R., Buttifant, M., Brown, D.J., Berlowitz, D.J. (2011). Assessment of breathing patterns and respiratory muscle recruitment during singing and speech in quadriplegia. Archives of Physical Medicine and Rehabilitation, 92(2), 250-256. doi: 10.1016/j.apmr.2010.10.032

Method and Results are clearly expressed. Appropriate use of tables and figures.

Line 82 – suggest removing the words “their respective respiratory muscles” and joining these two sentences.

Line 92 – suggest changing the word “chosen” to “eligible” and modifying this sentence:

“…volunteers with injury level below T11 were not eligible because there is minimal respiratory muscle impairment below this level of injury.”

Line 97 – typo – “vocal cord conditions”

Discussion and Implications are comprehensive and appropriate reflect on findings and study limitations in relation to previous research.

Possible typo on p12 line 359 “positive effects on other cognitive functions…”

Line 104 – remove the word “has”.

Lines 109-111

“Studies using alternative devices in measuring the mechanical activities of the respiratory muscles such as MMG are imperative to complement electromyography (EMG) assessments [33]. Some researchers have also suggested that a signal response that is inherently mechanical…”

Lines 114-115

“Two MMG sensors were attached to participants’ SCM and RA muscles to observe the accessory respiratory muscle activity while singing. These two muscles were chosen…”

Line 117

Plural error. Was there 1 or 2 MMG sensors place on SCM?

“MMG sensor on SCM was placed” or “MMG sensors on SCM were placed”

Line 127

“All participants were required to choose one song from each category.” Figure 2 shows only 1 song in each category though. Did they choose really?

Line 133

Suggest changing “follow the rhythm” to “follow the musical cues”

Line 138

What do you mean by: “Each song was recorded under three different trials”?

Line 144-145 What were the voice memo recordings used for?

Line 149 Suggest changing: “breathe-in and breathe-out activities were identified by referencing the voice recorded using Praat software” to “breathe-in and breathe-out activities were identified from the voice recordings using Praat software”

Line 152 “These timings were selected at the same section of the track for each participant.”

Didn’t each participant choose different songs though?

Results

Please add a results table and ensure to use exact p values in the results section (not just p0.05). Past tense also required. (eg. Line 173 “(LSD) test showed…”

It would be interesting to know if there were any differences in accessory respiratory muscle activation for people with different injury levels.

Discussion

Please use past tense and don’t present results/stats in discussion.

Line 180 – “MMG signal on the muscles while not singing is always lower compared to that of while singing (P 0.05).” Suggested revision: “MMG signals from the SCM and RA muscles were always higher when singing compared to when not singing.”

Line 192 – please clarify who you mean by “their”. Ie. “By activating accessory respiratory muscles like SCM, this may improve shortness of breath or dyspnoea for people with SCI.

Line 207 – please clarify what you mean by “follow through”.

Line 210 – the meaning of this sentence is unclear. Who are the “normal trained students”? Are these singing students? If so, this is a confusing comparison with classical singers.

“Among classical singers, abdominal muscles were also more active throughout singing session compared to those of normal, trained students.”

Are there any recommendations for which aspects of songs might be more beneficial for different levels of SCI? Ie. do higher level injured patients need to focus more on SCM or RA. Or are both equally beneficial? What about lower injury levels?

It would be good to have some clearer practice implications from this research if possible. Or if not, some clearer guidance for what further research is needed to provide clinical practice guidance.

Conclusion – please remove the unnecessary words “to conclude” in line 220

**********

6. PLOS authors have the option to publish the peer review history of their article (what does this mean?). If published, this will include your full peer review and any attached files.

If you choose “no”, your identity will remain anonymous but your review may still be made public.

**Do you want your identity to be public for this peer review?** For information about this choice, including consent withdrawal, please see our Privacy Policy.

Reviewer #1: No

Reviewer #2: **Yes: **Jeanette Tamplin

**********

[NOTE: If reviewer comments were submitted as an attachment file, they will be attached to this email and accessible via the submission site. Please log into your account, locate the manuscript record, and check for the action link "View Attachments". If this link does not appear, there are no attachment files.]

While revising your submission, please upload your figure files to the Preflight Analysis and Conversion Engine (PACE) digital diagnostic tool, https://pacev2.apexcovantage.com/. PACE helps ensure that figures meet PLOS requirements. To use PACE, you must first register as a user. Registration is free. Then, login and navigate to the UPLOAD tab, where you will find detailed instructions on how to use the tool. If you encounter any issues or have any questions when using PACE, please email PLOS at figures@plos.org. Please note that Supporting Information files do not need this step.

Attachment Submitted filename: Paper 3_PLOS One.docx

Attachment Submitted filename: PONE-D-23-07503 (reviewer tracked).pdf

Attachment Submitted filename: PLOS One - reviewer comments.docx

# Author comment: Author response to Decision Letter 0

**DOI:** 10.1371/journal.pone.0305940.r002

19 Mar 2024

A Point-by-Point Response to Reviewers’ Comments to the Author

No. Reviewer’s comment Response

1. Would muscle ‘activation’ be a better word choice than ‘performance’ in the title? Thank you for your suggestion. However, in our case ‘performance’ is better as we were using mechanomyography (MMG), not electromyography (EMG). 

EMG measures the electrical activity of the muscle during contraction generated by the brain. Meanwhile, MMG measures the mechanical vibrations produced by the muscle contraction.

2. Also, I’m not sure about the grammar of “singing at different song characteristics”. Maybe “songs with different musical parameters”? I have rearranged the title of the manuscript according to your recommendation from “Accessory Respiratory Muscles Performance During Singing at Different Song Characteristics Among People with Spinal Cord Injury” into “Accessory Respiratory Muscles Performance Among People with Spinal Cord Injury While Singing Songs with Different Musical Parameters”. 

3. Avoid use of pathologizing language such as “suffer from” (first line of abstract). More neutral or strengths-based language is preferred, such as “experience” or “live with”. The sentence is changed from “suffer from” into “experience”.

4. I’m also not sure about the phrase “rhythmic syllable” as one of the musical parameters of songs referred to throughout the paper. I think what you mean is “rhythmic complexity” or “syllable rhythmic complexity” if you want to keep the reference to syllables in there. Thank you for your input. The phrase “rhythmic syllable” is changed into “rhythmic complexity’” as pointed out by the reviewer, throughout the manuscript.

5. In the abstract it would be helpful to state the level of SCI. Eg. participants with SCI (C4 -T11 ASIA A or B). We have included the participants ASIA classification and injury level in the abstract. Thank you for the suggestion. 

6. Last line of abstract, suggest replacing “outcome” with “knowledge” – “Clinicians could benefit from this knowledge…” The word “outcome” has been replaced with the word “knowledge” in the last line of abstract.

7. Please state the exact number of songs that were characterized into the four categories. The exact number of songs is added into the manuscript in line 122. Also, the whole paragraph has been rearranged to ensure less confusion from readers.

8. Line 51-52 – suggest joining these 2 sentences for better flow: “This exercise is called respiratory muscle training (RMT) and includes exercises for inspiration, expiration or both of a certain duration and intensity to improve the strength and endurance of the muscles of respiration.”

 The 2 sentences in line 51-52 have been combined as mentioned by the reviewer.

9. Revise lines 65-66 based on comments above about the grammar of “singing at different song characteristics”. Maybe “songs with different musical parameters”? Also “rhythmic complexity” rather than “rhythmic syllable” Changes has been made accordingly in both cases.

10. Revise lines 69-70 for clarity of expression. Perhaps “rhythmic complexity refers to complexity of the rhythm of sung syllables. Rapid changes in the syllables of a song and complex or syncopated rhythm make it more difficult to sing.” Thank you for the correction. The lines 69-70 has been revised as stated by the reviewer.

11. Line 73 has a reference date which is not required in this referencing style. I have double checked the reference with ScienceDirect, and it still stated the same year as the one in this manuscript, which is 2013. 

12. Line 82 – suggest removing the words “their respective respiratory muscles” and joining these two sentences. Changes has been made accordingly.

13. Line 92 – suggest changing the word “chosen” to “eligible” and modifying this sentence:

“…volunteers with injury level below T11 were not eligible because there is minimal respiratory muscle impairment below this level of injury.” Line 92 has been revised as stated by the reviewer.

14. Line 97 – typo – “vocal cord conditions” Noted. The word “cords” has been changed into “cord”. 

15. Line 104 – remove the word “has”. The word “has” has been removed.

16. Lines 109-111

“Studies using alternative devices in measuring the mechanical activities of the respiratory muscles such as MMG are imperative to complement electromyography (EMG) assessments [33]. Some researchers have also suggested that a signal response that is inherently mechanical…” Thank you for pointing out the grammar mistakes. Changes has been made accordingly.

17. Lines 114-115

“Two MMG sensors were attached to participants’ SCM and RA muscles to observe the accessory respiratory muscle activity while singing. These two muscles were chosen…” Changes has been made accordingly.

18. Line 117

Plural error. Was there 1 or 2 MMG sensors place on SCM?

“MMG sensor on SCM was placed” or “MMG sensors on SCM were placed” There was only one sensor placed on SCM. Therefore, the correct sentence would be “MMG sensor on SCM was placed”. Changes has been made on line 117.

19. Line 127

“All participants were required to choose one song from each category.” Figure 2 shows only 1 song in each category though. Did they choose really? Participants were given a list of songs for each category, as shown in Appendix D. There are 48 songs on the list (as addressed in point no. 7). 

From that list, the participants will choose only one song for each category. 

Figure 2 only showed the four quadrants (or categories); (i) slow tempo easy low pitch (SEL), (ii) slow tempo easy high pitch (SEH), (iii) fast tempo hard low pitch (FHL) and (iv) fast tempo hard high pitch (FHH).

20. Line 133 

Suggest changing “follow the rhythm” to “follow the musical cues” Changes has been made as suggested.

21. Line 138

What do you mean by: “Each song was recorded under three different trials”? Each song was recorded for three attempts. The sentence has been changed to make sure less confusion from the readers.

22. Line 144-145 What were the voice memo recordings used for?

 The voice memo recordings were used together with Praat software to identify the exact moments of inspiration and expiration for data analysis.

23. Line 149 Suggest changing: “breathe-in and breathe-out activities were identified by referencing the voice recorded using Praat software” to “breathe-in and breathe-out activities were identified from the voice recordings using Praat software” Thank you for the suggestion. Changes has been made as stated by the reviewer.

24. Line 152 “These timings were selected at the same section of the track for each participant.”

Didn’t each participant choose different songs though? Yes, you are right. This is a mistake from my end. The sentence has been removed from the manuscript. 

25. Please add a results table and ensure to use exact p values in the results section (not just p0.05). Past tense also required. (eg. Line 173 “(LSD) test showed…” A new table (Table 2) has been added on the manuscript. The exact p values were also stated in the result section. The grammar error on Line 173 has also been corrected.

26. It would be interesting to know if there were any differences in accessory respiratory muscle activation for people with different injury levels. We did not perform any specific analysis to compare the accessory respiratory muscle activation between participants of different injury levels, thus are unable to provide a conclusive statement addressing this point. 

However, based on our crude observation, we do not see any difference in muscle performance between the different injury levels. 

We have added this in the discussion (line 217, page 10)

“Future research is needed to help clinicians in planning adequate singing therapy to strengthen the respiratory muscles in SCI, including investigation based on their levels of injury.”

27. Please use past tense and don’t present results/stats in discussion.

Line 180 – “MMG signal on the muscles while not singing is always lower compared to that of while singing (P 0.05).” Suggested revision: “MMG signals from the SCM and RA muscles were always higher when singing compared to when not singing.” Changes has been made accordingly.

28. Line 192 – please clarify who you mean by “their”. Ie. “By activating accessory respiratory muscles like SCM, this may improve shortness of breath or dyspnoea for people with SCI. Thank you for the suggestion. The sentence has been rearranged in the manuscript.

29. Line 207 – please clarify what you mean by “follow through”. From our experience during data collection, some participants found it harder to sing all the lyrics in songs categorized as hard rhythmic complexity. Some participants could not keep up with the lyrics due to the fast nature of the songs.

30. Line 210 – the meaning of this sentence is unclear. Who are the “normal trained students”? Are these singing students? If so, this is a confusing comparison with classical singers.

“Among classical singers, abdominal muscles were also more active throughout singing session compared to those of normal, trained students.” Thank you for notifying us with the mistake. Correction has been made accordingly.

31. please remove the unnecessary words “to conclude” in line 220 The word “to conclude” has been removed.

Attachment Submitted filename: Response to Reviewers Imran PlosOne.docx

# Editor report: Decision Letter 1

**DOI:** 10.1371/journal.pone.0305940.r003

**Authors:** Sahreen Anwar

9 Apr 2024

PONE-D-23-07503R1Accessory Respiratory Muscles Performance Among People with Spinal Cord Injury While Singing Songs with Different Musical ParametersPLOS ONE

Dear Dr. Ramli,

Thank you for submitting your manuscript to PLOS ONE. After careful consideration, we feel that it has merit but does not fully meet PLOS ONE’s publication criteria as it currently stands. Therefore, we invite you to submit a revised version of the manuscript that addresses the points raised during the review process.

==============================

**ACADEMIC EDITOR: **Please carefully consider the comments made by the reviewers. Make sure to get your manuscript formatted by a language expert.Overall, there is merit in the article but after incorporating these changes it will be more refined and presentable.==============================

Please submit your revised manuscript by May 24 2024 11:59PM. If you will need more time than this to complete your revisions, please reply to this message or contact the journal office at plosone@plos.org. When you're ready to submit your revision, log on to https://www.editorialmanager.com/pone/ and select the 'Submissions Needing Revision' folder to locate your manuscript file.

Please include the following items when submitting your revised manuscript:

- A rebuttal letter that responds to each point raised by the academic editor and reviewer(s). You should upload this letter as a separate file labeled 'Response to Reviewers'.
- A marked-up copy of your manuscript that highlights changes made to the original version. You should upload this as a separate file labeled 'Revised Manuscript with Track Changes'.
- An unmarked version of your revised paper without tracked changes. You should upload this as a separate file labeled 'Manuscript'.

If you would like to make changes to your financial disclosure, please include your updated statement in your cover letter. Guidelines for resubmitting your figure files are available below the reviewer comments at the end of this letter.

If applicable, we recommend that you deposit your laboratory protocols in protocols.io to enhance the reproducibility of your results. Protocols.io assigns your protocol its own identifier (DOI) so that it can be cited independently in the future. For instructions see: https://journals.plos.org/plosone/s/submission-guidelines#loc-laboratory-protocols. Additionally, PLOS ONE offers an option for publishing peer-reviewed Lab Protocol articles, which describe protocols hosted on protocols.io. Read more information on sharing protocols at https://plos.org/protocols?utm_medium=editorial-emailutm_source=authorlettersutm_campaign=protocols.

We look forward to receiving your revised manuscript.

Kind regards,

Sahreen Anwar, PhD Physical Therapy

Academic Editor

PLOS ONE

Journal Requirements:

Please review your reference list to ensure that it is complete and correct. If you have cited papers that have been retracted, please include the rationale for doing so in the manuscript text, or remove these references and replace them with relevant current references. Any changes to the reference list should be mentioned in the rebuttal letter that accompanies your revised manuscript. If you need to cite a retracted article, indicate the article’s retracted status in the References list and also include a citation and full reference for the retraction notice.

**Additional Editor Comments**

Thank you for submitting your work to PLOS ONE. There are some areas that need attention. Please get your article reviewed from a language expert, moreover, the technical language related to musicology must be simple and concise to enhance its readability.

[NOTE: If reviewer comments were submitted as an attachment file, they will be attached to this email and accessible via the submission site. Please log into your account, locate the manuscript record, and check for the action link "View Attachments". If this link does not appear, there are no attachment files.]

While revising your submission, please upload your figure files to the Preflight Analysis and Conversion Engine (PACE) digital diagnostic tool, https://pacev2.apexcovantage.com/. PACE helps ensure that figures meet PLOS requirements. To use PACE, you must first register as a user. Registration is free. Then, login and navigate to the UPLOAD tab, where you will find detailed instructions on how to use the tool. If you encounter any issues or have any questions when using PACE, please email PLOS at figures@plos.org. Please note that Supporting Information files do not need this step.

# Author comment: Author response to Decision Letter 1

**DOI:** 10.1371/journal.pone.0305940.r004

14 May 2024

Point-by-point responses to the editor comments:

1. Please get your article reviewed from a language expert. 

The manuscript has been proofread by a proofreader. A certificate from the proofreader is attached in the appendix below.

2. Please get your article reviewed from the technical language related to musicology must be simple and concise to enhance its readability. 

The manuscript has been proofread by two experts from music background: (1) Dr Marzelan Salleh, one of the co-authors of the manuscript from Music Department in Faculty of Creative Arts in Universiti Malaya, and (2) Dr Camellia Siti Maya Mohamed Razali from Music Department in Faculty of Human Ecology in Universiti Putra Malaysia.

3. Please review your reference list to ensure that it is complete and correct. If you have cited papers that have been retracted, please include the rationale for doing so in the manuscript text or remove these references and replace them with relevant current references. 

The reference list has been reviewed and several changes has been made in the DOI link. The changes were made because some of the DOI links were not working. This includes the link to references number 8, 9, 40, 41, 44, and 47. None of the references were retracted after reviewing the list.

4. While revising your submission, please upload your figure files to the Preflight Analysis and Conversion Engine (PACE) digital diagnostic tool, https://pacev2.apexcovantage.com/. PACE helps ensure that figures meet PLOS requirements. 

All figures from the manuscript have been uploaded in PACE website. The updated and corrected version of the figures have been uploaded during submission.

Attachment Submitted filename: Point by point responses.docx

# Editor report: Decision Letter 2

**DOI:** 10.1371/journal.pone.0305940.r005

**Authors:** Sahreen Anwar

10 Jun 2024

Accessory Respiratory Muscles Performance Among People with Spinal Cord Injury While Singing Songs with Different Musical Parameters

PONE-D-23-07503R2

Dear Dr. Ramli,

We’re pleased to inform you that your manuscript has been judged scientifically suitable for publication and will be formally accepted for publication once it meets all outstanding technical requirements.

Within one week, you’ll receive an e-mail detailing the required amendments. When these have been addressed, you’ll receive a formal acceptance letter and your manuscript will be scheduled for publication.

An invoice will be generated when your article is formally accepted. Please note, if your institution has a publishing partnership with PLOS and your article meets the relevant criteria, all or part of your publication costs will be covered. Please make sure your user information is up-to-date by logging into Editorial Manager at Editorial Manager® and clicking the ‘Update My Information' link at the top of the page. If you have any questions relating to publication charges, please contact our Author Billing department directly at authorbilling@plos.org.

If your institution or institutions have a press office, please notify them about your upcoming paper to help maximize its impact. If they’ll be preparing press materials, please inform our press team as soon as possible -- no later than 48 hours after receiving the formal acceptance. Your manuscript will remain under strict press embargo until 2 pm Eastern Time on the date of publication. For more information, please contact onepress@plos.org.

Kind regards,

Sahreen Anwar, PhD Physical Therapy

Academic Editor

PLOS ONE

Additional Editor Comments (optional):

Thank you for providing point to point response. we hope that the input from reviewers has contributed in improving the quality of your article.

# Editor report: Acceptance letter

**DOI:** 10.1371/journal.pone.0305940.r006

**Authors:** Sahreen Anwar

24 Jun 2024

PONE-D-23-07503R2 

PLOS ONE

Dear Dr. Ramli, 

I'm pleased to inform you that your manuscript has been deemed suitable for publication in PLOS ONE. Congratulations! Your manuscript is now being handed over to our production team.

At this stage, our production department will prepare your paper for publication. This includes ensuring the following:

* All references, tables, and figures are properly cited

* All relevant supporting information is included in the manuscript submission,

* There are no issues that prevent the paper from being properly typeset

If revisions are needed, the production department will contact you directly to resolve them. If no revisions are needed, you will receive an email when the publication date has been set. At this time, we do not offer pre-publication proofs to authors during production of the accepted work. Please keep in mind that we are working through a large volume of accepted articles, so please give us a few weeks to review your paper and let you know the next and final steps. 

Lastly, if your institution or institutions have a press office, please let them know about your upcoming paper now to help maximize its impact. If they'll be preparing press materials, please inform our press team within the next 48 hours. Your manuscript will remain under strict press embargo until 2 pm Eastern Time on the date of publication. For more information, please contact onepress@plos.org.

If we can help with anything else, please email us at customercare@plos.org.

Thank you for submitting your work to PLOS ONE and supporting open access. 

Kind regards, 

PLOS ONE Editorial Office Staff

on behalf of

Dr. Sahreen Anwar 

Academic Editor

PLOS ONE
